# The localization of amyloid precursor protein to ependymal cilia in vertebrates and its role in ciliogenesis and brain development in zebrafish

**DOI:** 10.1038/s41598-021-98487-7

**Published:** 2021-09-27

**Authors:** Jasmine Chebli, Maryam Rahmati, Tammaryn Lashley, Brigitta Edeman, Anders Oldfors, Henrik Zetterberg, Alexandra Abramsson

**Affiliations:** 1 Department of Psychiatry and Neurochemistry, Institute of Neuroscience and Physiology, The Sahlgrenska Academy, University of Gothenburg, 41345 Gothenburg, Sweden; 2 Department of Clinical and Movement Neurosciences, Queen Square Brain Bank for Neurological Disorders, Queen Square Institute of Neurology, University College London, London, UK; 3 Department of Neurodegenerative Disease, UCL Institute of Neurology, Queen Square, London, UK; 4 Department of Laboratory Medicine, University of Gothenburg, Gothenburg, Sweden; 5 Clinical Neurochemistry Laboratory, Sahlgrenska University Hospital, Mölndal, Sweden; 6 UK Dementia Research Institute, London, UK

**Keywords:** Ciliogenesis, Cellular neuroscience, Neuroscience, Alzheimer's disease, Neurodegeneration

## Abstract

Amyloid precursor protein (APP) is expressed in many tissues in human, mice and in zebrafish. In zebrafish, there are two orthologues, Appa and Appb. Interestingly, some cellular processes associated with APP overlap with cilia-mediated functions. Whereas the localization of APP to primary cilia of in vitro-cultured cells has been reported, we addressed the presence of APP in motile and in non-motile sensory cilia and its potential implication for ciliogenesis using zebrafish, mouse, and human samples. We report that Appa and Appb are expressed by ciliated cells and become localized at the membrane of cilia in the olfactory epithelium, otic vesicle and in the brain ventricles of zebrafish embryos. App in ependymal cilia persisted in adult zebrafish and was also detected in mouse and human brain. Finally, we found morphologically abnormal ependymal cilia and smaller brain ventricles in *appa*^*−/−*^*appb*^*−/−*^ mutant zebrafish. Our findings demonstrate an evolutionary conserved localisation of APP to cilia and suggest a role of App in ciliogenesis and cilia-related functions.

## Introduction

Amyloid precursor protein (APP) is a ubiquitously expressed type-1 transmembrane protein that, together with the APP-like protein 1 and -2 (APLP1, APLP2), comprises the APP protein family in humans and mice. In contrast, zebrafish have, due to the partial third genome duplication, two APP orthologues, Appa and Appb, that share high sequence similarity^1^. Zebrafish also express the two other App-family members Aplp1 and Aplp2. The mild phenotypes of mice with single mutations suggest redundancy between App family members^2^. In addition to their various splice forms, they are all post-translationally modified through proteolytic processing^3^. Although the physiological relevance of the fragments generated is not fully understood, one of these, the amyloid-beta peptide (Aβ) originating from the transmembrane domain of the APP protein, is the main component of brain amyloid plaques in Alzheimer’s disease (AD)^3,4^. Beyond its pathological involvement, studies on APP have revealed essential physiological functions including neurogenesis^5,6^, neurite outgrowth^7,8^, axonal transport^9–13^, adhesion properties^14–16^, synapse formation^17–19^, neuronal migration^8,20,21^, mitochondria-associated ER membranes (MAMs) activity and cholesterol metabolism^22,23^. Nevertheless, the involvement of each APP family member in these processes remains unclear, since redundancy makes it difficult to unravel the contribution of a specific protein^24^. Although the molecular mechanisms behind the APP-related processes are yet to be determined, accumulating evidence support that APP orchestrates cellular processes through receptor-like interactions with both inter- and intra-cellular signaling molecules^8^.

The cilium is a highly conserved organelle across species, which contributes to a wide range of cellular processes^25^. Cilia can broadly be categorized into motile and non-motile. Non-motile cilia include primary cilia, which are ubiquitously expressed on most cells as a single short antenna-like structure, and sensory cilia, that are only expressed by specific cells. Primary cilia are enriched in receptors and sites for inter-cell signaling transduction and are notably implicated in cell division, autophagy, midbrain development, memory and learning abilities^26^. As for the sensory cilia, they are notably found in the otic vesicle as stereocilia and kinocilia. Motile cilia are present on cells involved in fluid movement including the epithelium of the respiratory tract and the ependymal layer of the brain ventricles. Ependymal cells are derived from radial glial cells and when fully differentiated are decorated with tufts of motile cilia anchored with roots at the apical cellular membrane^25,27^. The coordinated periodic beating of the cilia participate in the generation of cerebrospinal fluid (CSF) flow within ventricle cavities^28^. Circulation of CSF is believed to facilitate transfer of signaling molecules and removal of metabolic waste products to prevent accumulation of neurotoxic residues in the brain parenchyma^29–31^.

There are several findings supporting a connection between APP and cilia. First, part of the wide range of cilia-mediated functions overlap with processes linked to APP, *e.g.*, cognitive impairment^32^, differentiation of neurons^33^, formation of corpus callosum^32,34^, neuronal migration^35–37^ and sensing of guidance molecules^38^. Second, overexpression of APP impairs primary cilia both in a mouse AD model and in individuals with Down syndrome, harboring three copies of *APP*^39,40^. The latter is also associated with decreased CSF flow and accumulation of CSF (hydrocephalus), two phenotypes commonly associated with defects in motile cilia^41^. Finally, APP has been shown to localize to primary cilia in vitro and Aβ exposure results in reduced cilium length^42^. Taken together, these findings warrant further investigations of the role of APP in both motile and non-motile cilia.

In the present study, we address the presence of APP in motile and non-motile (sensory) cilia and its possible functions using zebrafish, mouse and human samples. We found that the zebrafish App homologues are expressed by ciliated cells and become localized at the membrane of cilia in the otic vesicle, the nasal epithelium, and the brain ventricles of zebrafish embryos. The presence of App in ependymal cilia persisted in adult zebrafish and was also detected in the ependymal cells of mouse and human brains. In addition, we show that zebrafish embryos with mutations in both *app* paralogues (*appa*^*−/−*^*appb*^*−/−*^*)* have morphologically abnormal ependymal cilia and smaller brain ventricles compared with wild-type siblings.

## Results

### *Appa* and *appb* mRNA expression patterns at the brain ventricular limits

**Figure 1:**
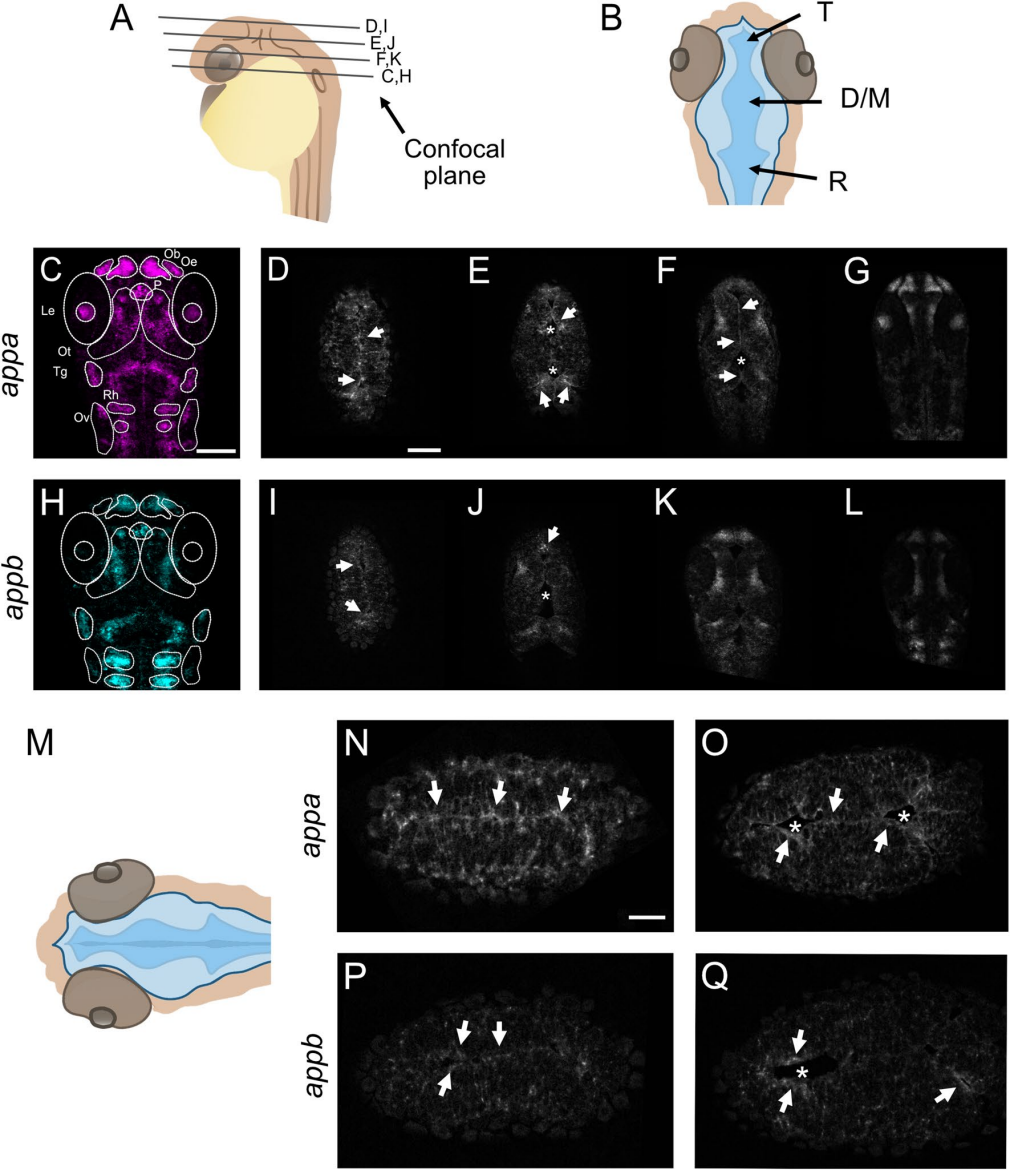
Expression pattern of *appa* and *appb* mRNA. (**A**,**B**) Schematic representations of head and ventricle morphology of a 30 hpf zebrafish larvae, lateral (**A**) and dorsal (**B**) view. (**C**,**H**) Whole-mount fluorescent in situ of *appa* (**C**) and *appb* (**H**) in 30 hpf WT zebrafish larvae. Single focal planes, dorsal to ventral, of whole-mount larvae of *appa* (**D**–**G**) and *appb* (**I**–**L**) probe. (**M**) Schematic view of focal plane of the dorsal area of the brain ventricle. (**N**–**Q**) Single focal plane at high magnification (40×) of *appa* (**N,O**) and *appb* (**P,Q**) probes. *T* telencephalic ventricle, *D/M* diencephalic/mesencephalic ventricle, *R* rhombencephalic ventricle, *Ob* olfactory bulb, *Oe* olfactory epithelium, *P* pituitary gland, *Le* lens, *Ot* optic tectum, *Tg* trigeminal ganglia, *Rh* rhombomeres, *Ov* otic vesicle. Magnification: (**C**–**L**) = 20×, (**N**–**Q**) = 40×. Scale bar: (**C**) = 100 µm, (**D**) = 50 µm, (**N**) = 25 µm. *Indicates ventricular space and white arrows highlight *appa* and *appb* expression at the ventricular borders.

The zebrafish *app* genes, *appa* and *appb,* are expressed in the CNS, and have both distinct and shared expression patterns^1,14^. Due to the lack of specific antibodies, we used fluorescent whole mount in situ hybridization to increase the cellular resolution of *appa* and *appb* mRNA expression in areas with motile cilia on 30 hpf wild-type larvae zebrafish (Fig. 1). Consistent with previous studies, we observed *appa* mRNA expression in the lens, the olfactory bulb and epithelium, in the trigeminal ganglia and in the otic vesicle. (Fig. 1C). Similarly, the *appb* mRNA expression signal corroborated previous data on *appb* mRNA expression^1^ in the olfactory and otic vesicle epithelia (Fig. 1H).

In addition, both *appa* (Fig. 1C–G and high magnification Fig. 1N,O) and *appb* (Fig. 1H–L,P,Q) mRNA signals labelled cells lining the diencephalic ventricle both in the dorsal and ventral areas. Negative controls did not show any specific signal (Supplementary file 1). Together, these results show expression of *appa* and *appb* in areas with ciliated cells, including cells lining brain ventricles, otic vesicle and olfactory organ, thus suggesting a possible role of App in cilia formation and function.

### App protein is localized to cilia of the olfactory sensory neurons and otic vesicle in zebrafish larvae

The expression of both *appa* and *appb* in ciliated cells made us ask if the proteins become distributed out to the cilia. The zebrafish olfactory epithelium and the otic vesicle comprise ciliated cells and are regions where both *appa* and *appb* mRNAs are expressed. To address if Appa and Appb become localized to these cilia, we performed immunofluorescent staining on zebrafish larvae.

#### Olfactory sensory neuron cilia

**Figure 2:**
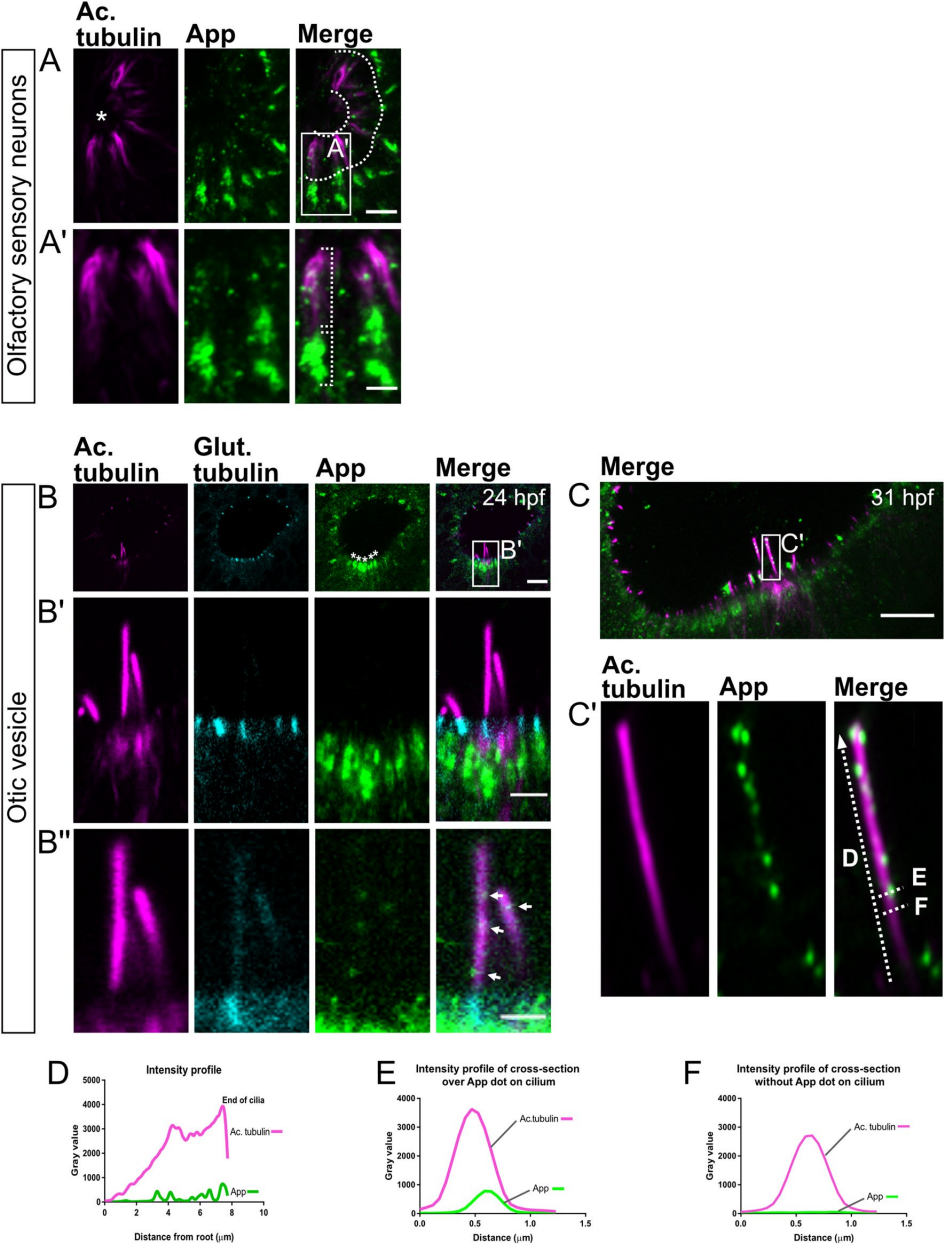
Localization of App protein to cilia of the olfactory sensory neurons and otic vesicle in 31 hpf larvae. Cilia as shown by immunostaining for acetylated tubulin (magenta) and App (green) of the olfactory sensory neurons in the nose epithelium (**A**) and the otic vesicle (**B**–**C**). In (**A**), dotted lines demarcate the cilia from the nasal cavity (see asterisk). (**A’**) App (green) is found along the cilia and accumulating at their base. Otic vesicle of 24 hpf (**B**) and 31 hpf larvae (**C**). In (**B**), glutamylated tubulin (cyan) highlights the base of the cilia outlined by acetylated tubulin staining (magenta). (**B**) Overview of the kinocilia and stereocilia of the otic vesicle. The white asterisks indicate accumulation of App (green) at the base of the cilia bundles. (**B’**) Magnification of cilia outlined in (**B**). (**B’’**) Increased intensity of the green channel to detect App (arrows) in kinocilia. Otic vesicle in 31hpf zebrafish larvae (**C**) with close-up (**C’**) showing App puncta (green) along the kinocilia. Intensity profiles of acetylated tubulin (magenta) and App (green) staining from the kinocilia (**D**–**F**). In (**D**), the intensity profile of the whole length of the kinocilium is plotted whereas profiles of cross-section lines are plotted with a visible App puncta (**E**) and without (**F**). The dotted lines (**C’**) indicate the kinocilium and cross-sections. Magnification: (**A**–**C**) = 40×. Scale bar: (**A**) = 5 µm, (**B**) = 10 µm, (**B’**) = 4 µm, (**B’’**) = 2 µm, (**C**) = 10 µm.

We used the Y188 antibody, binding to a conserved epitope in the C-terminal end of human, mouse, and zebrafish App (Fig. 6C), in combination with the anti-acetylated tubulin antibody, labelling microtubule structures of cilia. Immunofluorescent co-labelling detected a punctate App signal in the heavily ciliated olfactory epithelium area at 30 hpf (Fig. 2A). However, while the resolution of the images did not allow distinction between each cilium, App signal seemed to localize to most of them. In addition to the cilium, App expression was also found at the base of these motile cilia (Fig. 2A’).

#### Otic vesicle cilia

Similar to the olfactory neurons, high accumulation of App was noted at the base of the cilia in the otic vesicle. In zebrafish, hair cells of the otic vesicle have two types of cilia, a long single kinocilium and a bundle of shorter stereocilia^43^. The immunofluorescent staining revealed App expression in both types of cilia at early time points in the larvae development (Fig. 2B,C). Staining of 24 hpf larvae with glutamylated tubulin, highlighting the cilia basal bodies, clearly showed an App signal within the hair cells and close to the basal body (Fig. 2B,B’,B’’). App expression became more distinct at 30 hpf (Fig. 2C,C’). Plots of the intensity profile of App (green) and acetylated tubulin (magenta) showed a punctate distribution of App throughout the kinocilium (Fig. 2D), which supports that App localizes to the cilium membrane (Fig. 2E). No signal was detected in the intensity profile in the absence of App puncta (Fig. 2F). Together, these data show expression of App in cilia and ciliated cells of the otic vesicle and olfactory bulb and indicate that App is located at the cilium membrane.

### App localizes to cilia decorating the brain ventricle surface of zebrafish

**Figure 3:**
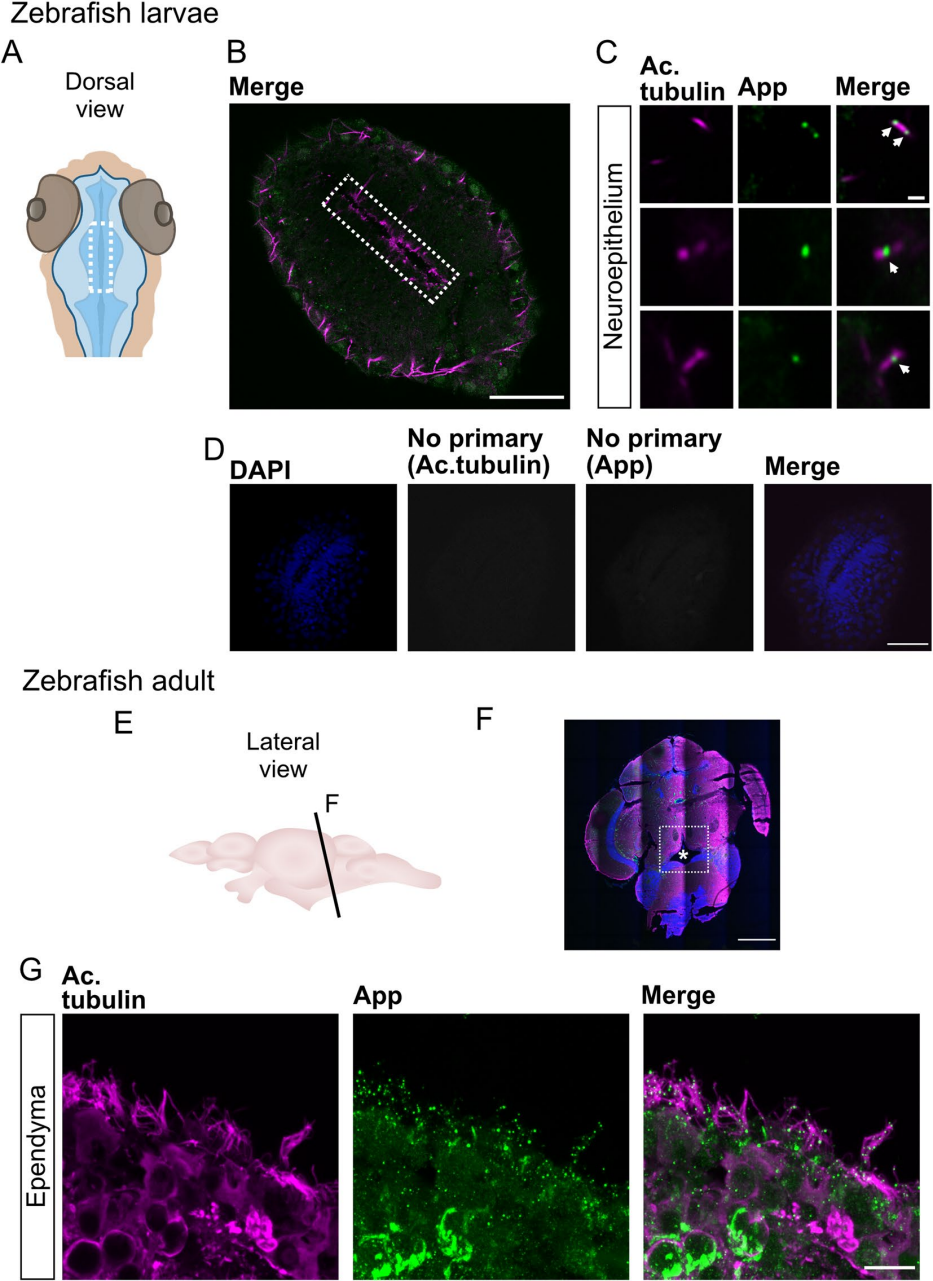
App localizes to the cilia decorating the ventricle of larvae and ependymal cells in adult zebrafish. (**A**) Schematic representations of head and ventricle morphology in 30 hpf zebrafish larvae, dorsal view. (**B**) Dorsal view of ventricle immunostained for App (green) and acetylated tubulin (magenta) in 30 hpf WT zebrafish larvae. (**C**) Close-up of cilia (magenta) and App (green). (**D**) Negative controls for immunofluorescence with secondary antibodies without anti-acetylated tubulin and anti-App primary antibodies. Cell nuclei stained with DAPI (blue). (**E**) Schematic outline of adult zebrafish brain, lateral view. (**F**–**G**) Coronal section of adult zebrafish brain and the central canal (see asterisk). Cell nuclei labeled with DAPI (blue), acetylated tubulin (magenta), App (green). (**G**) Ependymal motile cilia (magenta) of the central canal with App (green) accumulation along cilia. Magnification: (**B**,**F**) = 10×, (**D**) = 40×, (**C**,**G**) = 60×. Scale bar: (**B,D**) = 50 µm, (**C**) = 1 µm, (**F**) = 500 µm, (**G**) = 10 µm.

As APP was previously shown to be expressed by the ependymal cells in rodents and in humans^44–46^, we explored App expression by ependymal cells and App localization at their cilia in larvae and adult zebrafish (Fig. 3). At 30 hpf, the brain ventricles are inflated and the differentiation of motile cilia in the most ventral and dorsal regions have just started but do not yet contribute to directional CSF flow^47^. This facilitates whole-mount imaging and measurement of single cilium. Using the same combination of antibodies (anti-APP (Y188) and anti-acetylated tubulin) as above, we could detect App-positive puncta along the acetylated tubulin signal in most cilia (Fig. 3B,C). To address if App localization to cilia is maintained into adulthood, we performed immunofluorescent staining on coronal sections of adult zebrafish brains using antibodies detecting App (Y188) and acetylated tubulin to label cilia. Our results showed that consistent to larvae, App was distributed to ependymal cilia in the adult brain. In contrast to larvae, ependymal cells in adult individuals were covered with multiple motile cilia. Cryosections of adult zebrafish brain revealed dense cilia tufts with App-positive staining at the apical side of the ependymal cells (Fig. 3F,G). Furthermore, App was also expressed by ependymal cells, similarly to what has previously been described in rodents and humans (Fig. 3G). Negative controls did not show any cilia-specific staining (Fig. 3D). No signal was detected in adult zebrafish samples for App in the negative control staining (absence of primary antibody Y188) or in the presence of rabbit IgG and secondary antibody (Supplementary file 2).

### Conserved localization of APP in ependymal cilia in mouse and human brains

**Figure 4:**
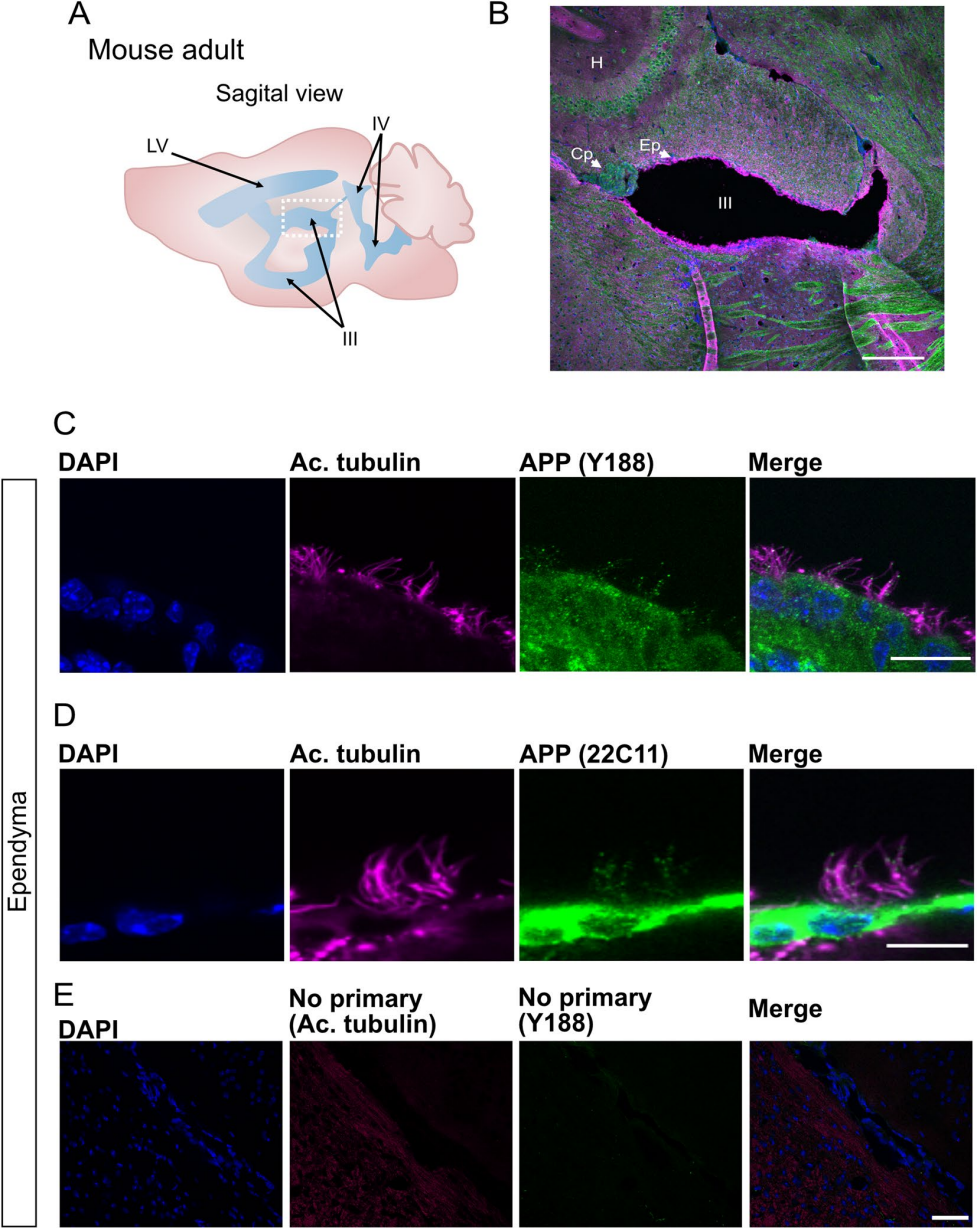
APP is localized to the ependymal cilia in adult mouse. (**A**) Schematic representation of adult mouse brain ventricular system, sagittal view. (**B**) Overview of sagittal section from adult mouse brain and the third ventricle (see dotted square in (**A**)) for cell nuclei stained with DAPI (blue), acetylated tubulin (magenta), APP (green). (**C**–**D**) Close-up of ependymal cells and their cilia tufts (magenta) and APP expression with anti-APP Y188 antibody (**C**) and 22C11 antibody (**D**). (**E**) Negative controls of immunofluorescence staining without primary antibodies and only with the corresponding secondary antibodies. For cell nuclei with DAPI (blue). *LV* lateral ventricle, *III* third ventricle, *IV* fourth ventricle, *H* hippocampus, *Cp* choroid plexus, *Ep* ependyma. Magnification: (**B**) = 10×, (**E**) = 40×, (**C,D**) = 60×. Scale bar: (**B**) = 200 µm, (**C**,**D**) = 10 µm, (**E**) = 50 µm.

APP is also localized to ependymal cilia in mice and humans. We performed immunostaining on mouse brain sections using two antibodies directed to APP, Y188 binding to the C-terminal intra-cellular domain and 22C11 detecting the E1 domain of the N-terminal region (Fig. 6C), together with anti-acetylated tubulin. The ependymal motile cilia were easily observed in the third ventricle of the brain sagittal section (Fig. 4A,B). Congruent with our results on adult zebrafish brains, we detected strong APP expression with both antibodies throughout the ependymal cells layer and punctate APP staining (Y188 see Fig. 4C and 22C11 see Fig. 4D) overlapping with acetylated tubulin-positive cilia. Interestingly, APP expression by the choroid plexus cells was detectable (Fig. 4B). Negative controls for primary antibodies were performed and showed no or weak signal (Fig. 4E).

**Figure 5:**
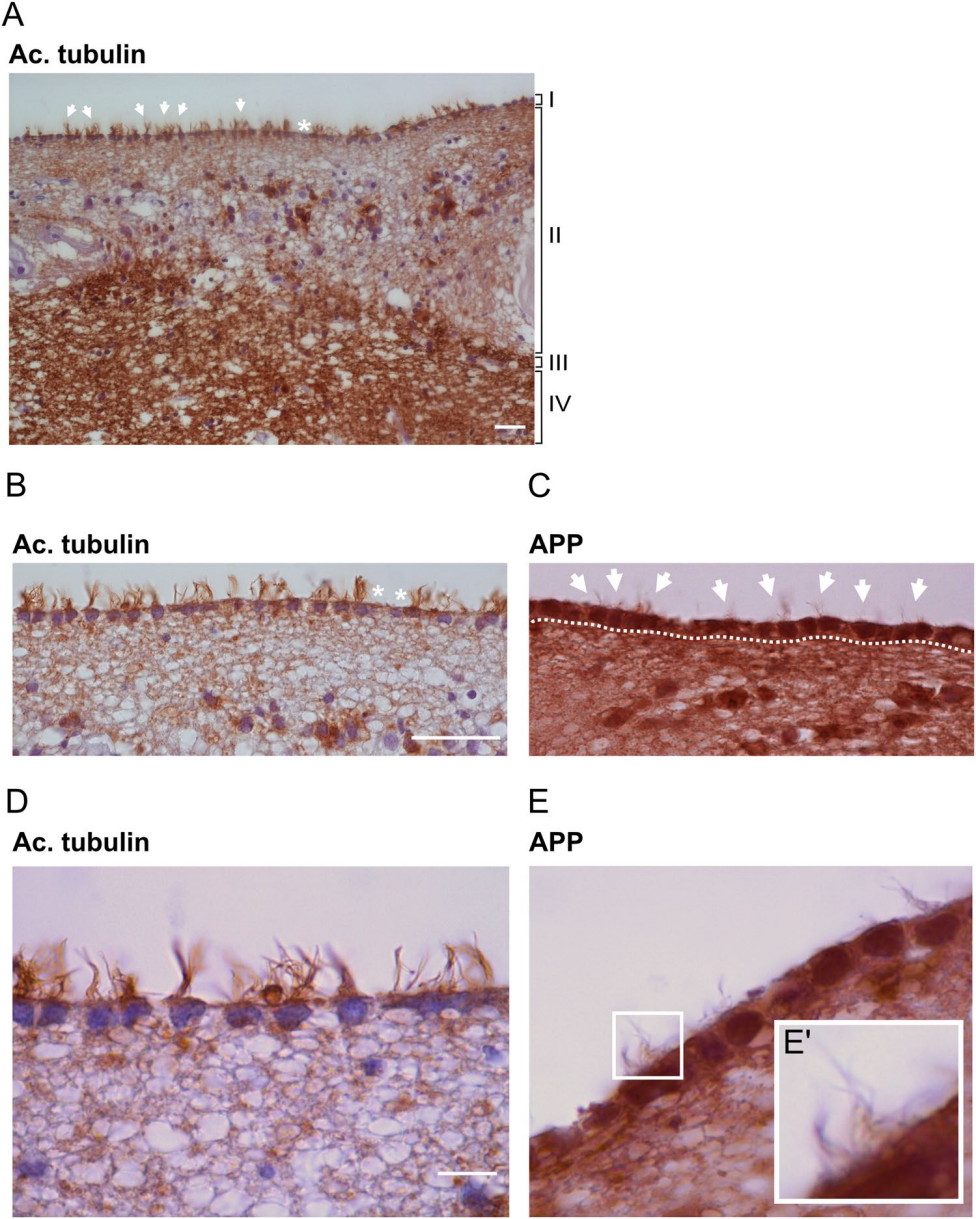
APP is localized to human ependymal cilia. (**A**) Brightfield overview of a human brain section of the caudate nucleus immunostained with an anti-acetylated tubulin antibody reveals the different cellular layers (I–IV): (I) ependymal layer with motile cilia orienting towards the ventricle lumen, (II) cellular extensions connecting the ependymal cells, (III) cellular layer dense in astrocytes, (IV) brain parenchyma. (**B**–**E**) Higher magnifications of the ependymal layer show clear cilia (acetylated tubulin (**B**,**D**)) and APP (Y188 antibody (**C**,**E**)) accumulation within ependymal cells and along ependymal cilia. (**E’**). Arrows highlight ependymal cilia tufts in the ventricular lumen. White asterisks indicate broken or absent cilia. Dotted lines delimitate the ependymal cell layer. Magnification: (**A**) = 20×, (**B**,**C**) = 40×, (**D**,**E**) = 100×. Scale bar: (**A**) = 5 µm, (**B**) = 10 µm, (**D**) = 2 µm.

In the human brain, acetylated tubulin staining allowed separation of cellular layers of the caudate nucleus and identification of acetylated tubulin-positive cilia of the ependymal cell layer lining the lateral ventricle (Fig. 5A,B,D). However, while many ependymal cells had intact cilia, many were found broken and dislocated from their cell (Supplementary file 3).

To support the presence of APP in ependymal cilia, brain serial sections of the caudate nucleus were incubated with horseradish peroxidase (HRP)-conjugated Y188 or A8717 antibodies, both recognizing the C-terminal domain of APP. Similarly to our results obtained in mouse and zebrafish brains, brightfield images confirmed strong APP expression in the ependymal cells and, upon higher magnification, in ependymal cilia (Fig. 5C,E, Supplementary file 3). In contrast to zebrafish and mouse, APP in human ependymal cilia was evenly distributed and was not detected as puncta.

In summary, these results show that the expression of APP in the ependymal cells and their cilia are conserved between species as far apart as zebrafish, mice, and humans.

### Generation of *appa* and *appb* double mutant zebrafish

**Figure 6:**
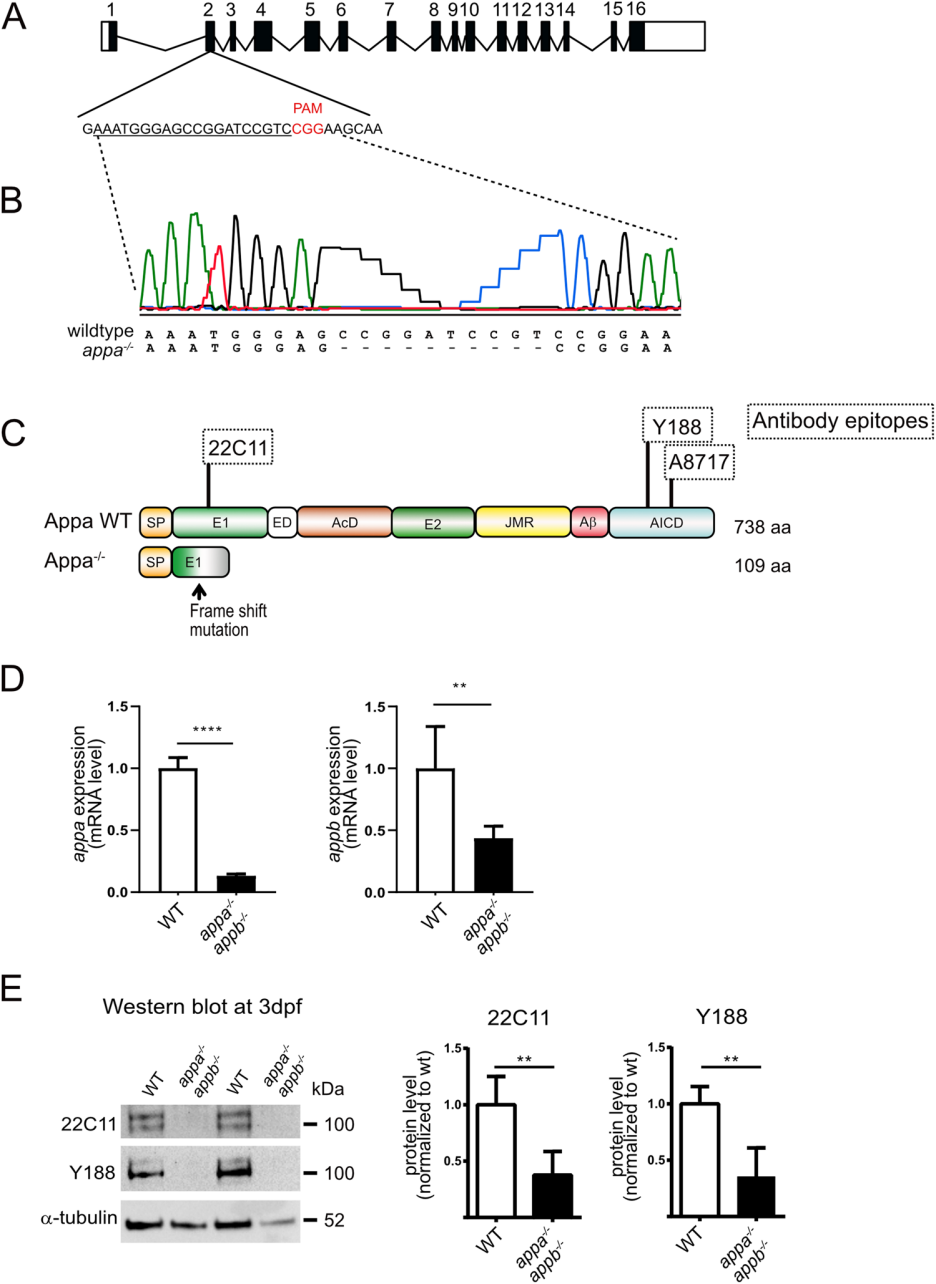
Generation of appa^*−/−*^ and analysis of appa^*−/−*^appb^*−/−*^ double mutant zebrafish. (**A**) Schematic outline of the *appa* gene with exons (black box) and UTR regions (white box). sgRNA used to target exon 2 with protospacer adjacent motif (PAM) in red and the sgRNA target sequence underlined. (**B**) Sanger sequencing chromatogram of the exon 2 region targeted in wild-type zebrafish. The *appa*^*−/−*^ mutant sequence is given below with dash indicating deleted nucleotides. (**C**) Schematic drawing of the wild-type Appa protein (738 aa) with epitopes of antibodies (dotted squares) used above and the hypothetical truncated Appa (109 aa) protein produced in *appa* mutant below. (**D**) qPCR quantification of *appa* and *appb* mRNA levels in wild-type and *appa*^*−/−*^*appb*^*−/−*^ mutants at 24 hpf. (**E**) Western blot of 3 dpf whole larvae zebrafish with antibodies against 22C11 and App (Y188). Alpha-tubulin is used as loading control. Blots cropped from the same original gel and grouped. Quantification of band intensity are shown relative to control. Data are reported as mean ± SD. ** ρ < 0.05, **** ρ < 0.001. qPCR n = 5, WB n = 3. *SP* signal peptide, *E1* extracellular domain, *ED* extension domain, *AcD* acidic domain, *E2* extracellular domain 2, *JMR* juxtamembrane region, *Aβ* amyloid beta, *AICD* amyloid intracellular domain.

In contrast to humans and mice, zebrafish have two *APP* orthologues, *appa* and *appb* (together designated *app*). The zebrafish *appb*^*26_2*^ mutant, carrying a frame shift deletion of five base pairs at the end of exon two, which introduce a premature stop codon, was generated and described by our lab previously^14^. However, to investigate the requirement of both App proteins in ciliogenesis, we used the CRISPR/Cas9 method to generate mutations in the zebrafish *appa* gene (Fig. 6A). A mutation was identified in exon 2 (Fig. 6A), and Sanger sequencing confirmed a deletion of 10 nucleotides (Fig. 6B). The mutation resulted in a premature stop codon predicted to give rise to a protein truncation at amino acid 109 (Fig. 6C). The *appa* mutant allele was outcrossed into the AB background until generation F4 and then bred with the *appb*^*−/−*^ to generate the double mutant *appa*^*−/−*^*appb*^*−/−*^ zebrafish line. The *app* mutant zebrafish were healthy and fertile and did not show any gross morphological phenotypes. qPCR analysis of genes expression showed very low *appa* and *appb* mRNA levels in the double mutant fish line (Fig. 6D). Western blot analysis using the Y188 and 22C11 antibodies with epitopes in the intracellular and extracellular domain, respectively, showed decreased protein levels in *app* double-mutant larvae (Fig. 6E and Supplementary file 4). Both antibodies are likely cross-reacting with Aplp2 since the epitope sequences are highly similar. These data show that the introduced mutation in *appa* resulted in a significant decrease of both transcription and translation of the Appa protein indicating that the mutation gives rise to a loss-of-function mutation.

### Longer brain ventricle cilia in ***appa***^*−/−*^***appb***^*−/−*^ larvae

**Figure 7:**
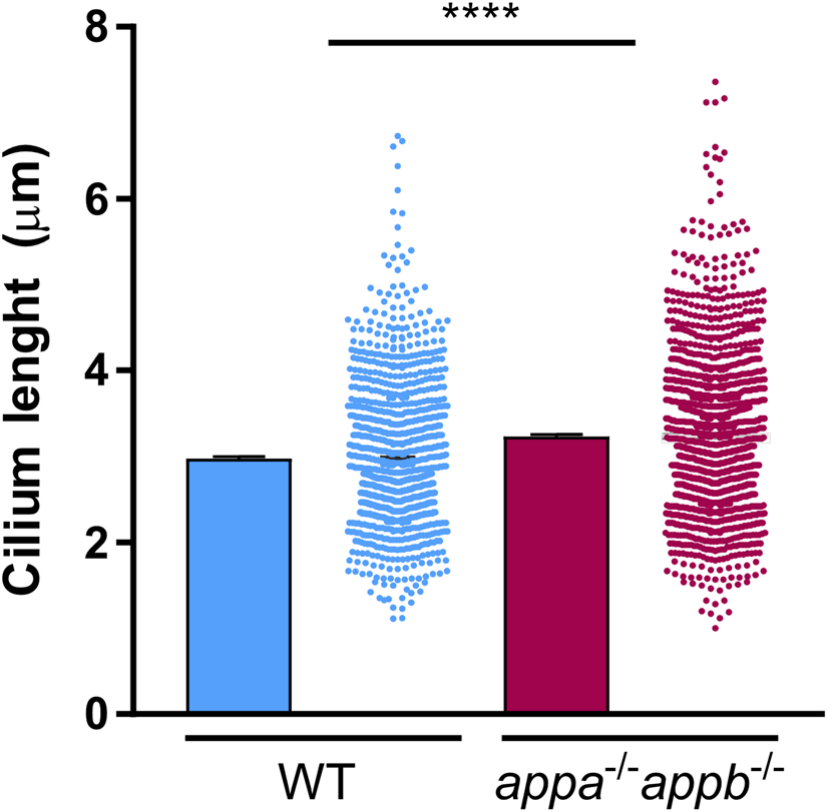
Longer cilia of dorsal brain ventricle neuroepithelium in *appa*^*−/−*^*appb*^*−/−*^ larvae zebrafish*.* At 30hpf, *appa*^*−/−*^*appb*^*−/−*^ exhibit longer diencephalic/mesencephalic ventricle cilia than WT. Data are reported as mean ± SEM. **** ρ < 0.001. n = 10 WT (1091 cilia), 16 *appa*^*−/−*^*appb*^*−/−*^ (1511 cilia).

The conserved distribution of APP in brain ventricle cilia prompted us to address the requirement of App during ciliogenesis. We measured the length of cilia in the midbrain ventricle detected by acetylated tubulin immunostaining signal in both *appa*^*−/−*^*appb*^*−/−*^ double mutants and wild-type larvae at 30 hpf. At this stage, the cilia delineating the dorsal and ventral parts of the diencephalic ventricles are not yet motile^47^. A 3D-region of interest (ROI) was used to measure cilium length. The ROI was established from the dorsal part of the midbrain ventricle to the ventricular space at a depth of around 25 μm. To our surprise, we found that the ependymal cilia in the ROI were significantly longer in *appa*^*−/−*^*appb*^*−/−*^ mutants compared with wild-type larvae (Fig. 7), which was confirmed by frequency distribution (Supplementary file 5).

### Integrity of ependymal cilium axoneme and microtubule doublets in motile brain ependymal cilia in ***appa***^*−/−*^***appb***^*−/−*^ mutant adult zebrafish

**Figure 8:**
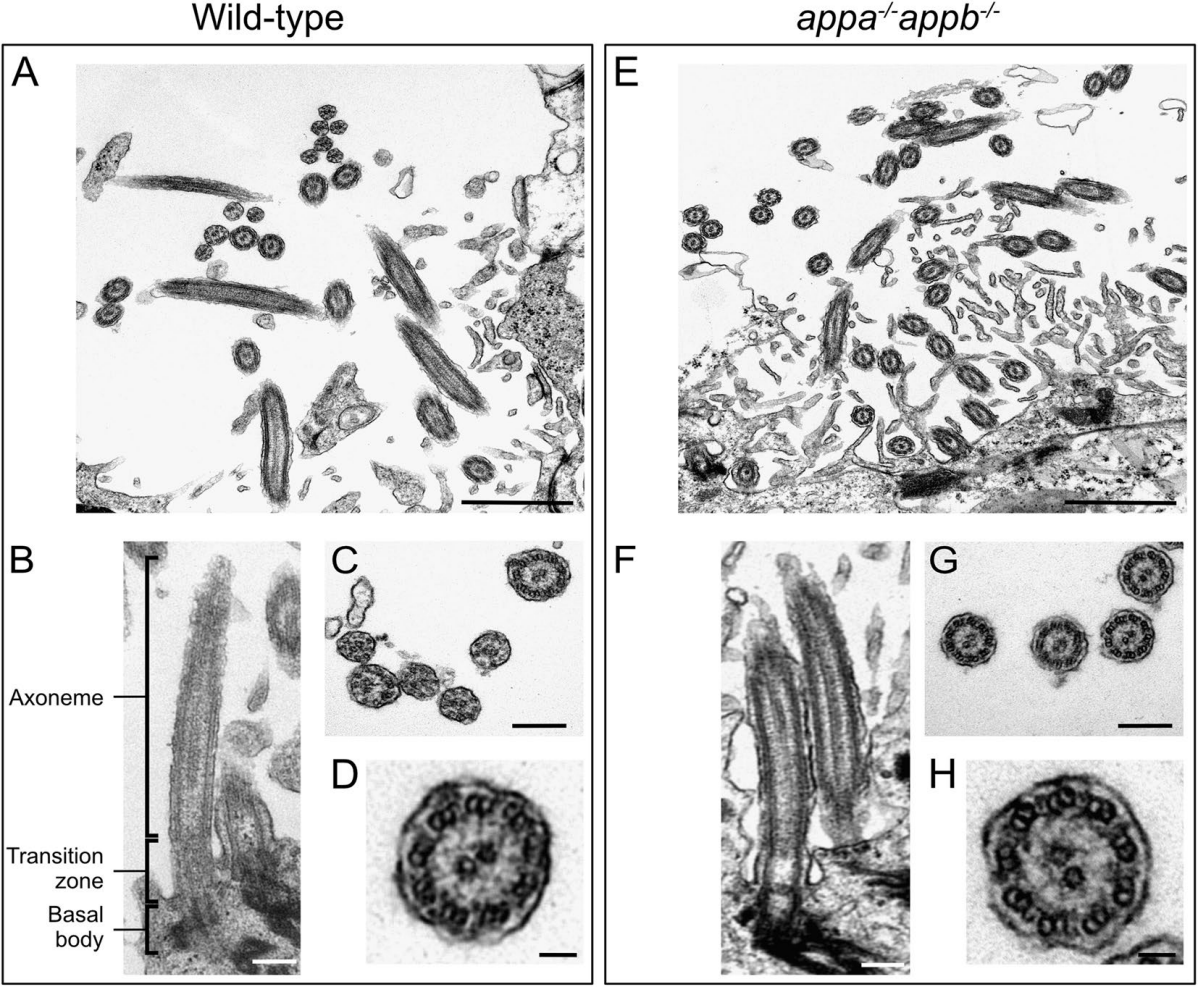
Structural integrity of ependymal cilia in WT and *appa*^*−/−*^*appb*^*−/−*^ zebrafish. Transmission electron microscopy of adult zebrafish ependymal cilia of WT (**A**–**D**) and *appa*^*−/−*^*appb*^*−/−*^ mutant (**E**–**H**) adult zebrafish. (**A**,**E**) Overview of ependymal cilia of the central canal. (**B**,**F**) Longitudinal view on the axoneme of the cilia composing its core, the transition zone including the ciliary pit between the cilia core and the cellular membrane and the basal body containing the cilia centrioles, highlighted with increased signal. In (**C**,**G**), cross-sections of cilia. (**D**–**H**) Zoom on cross-section of individual cilia showing (9 + 2) microtubule doublet organization. Scale bar: (**A**,**E**) = 1 µm, (**B**,**C**,**F**,**G**) = 200 nm, (**D**,**H**) = 50 nm.

Emerging from the basal body is the axoneme, which forms the core of the cilium. First described in the early 1950s with electron microscopy, axonemes are composed of nine microtubule doublets at the periphery (9 + 0)^48^. In some cilia, an additional central doublet is expressed (9 + 2), allowing cilia to generate and regulate movement^49,50^. This central microtubule doublet is found in motile ependymal cilia (9 + 2). To better characterize the ciliary ultrastructure of App-deficient zebrafish, we performed transmission electron microscopy (TEM) analysis of ependymal cells in adult zebrafish brains. TEM revealed a normal (9 + 2) axoneme in the cross-sections of ependymal cilia of WT (n = 3 zebrafish, 96 cilia) brain ventricle (Fig. 8A–D). In *appa*^*−/−*^*appb*^*−/−*^ zebrafish (n = 4 zebrafish, 123 cilia), ependymal cilia showed normal (9 + 2) axonemes (Fig. 8E–H).

### The ***appa***^*−/−*^***appb***^*−/−*^ double mutants exhibit smaller diencephalic ventricle

**Figure 9:**
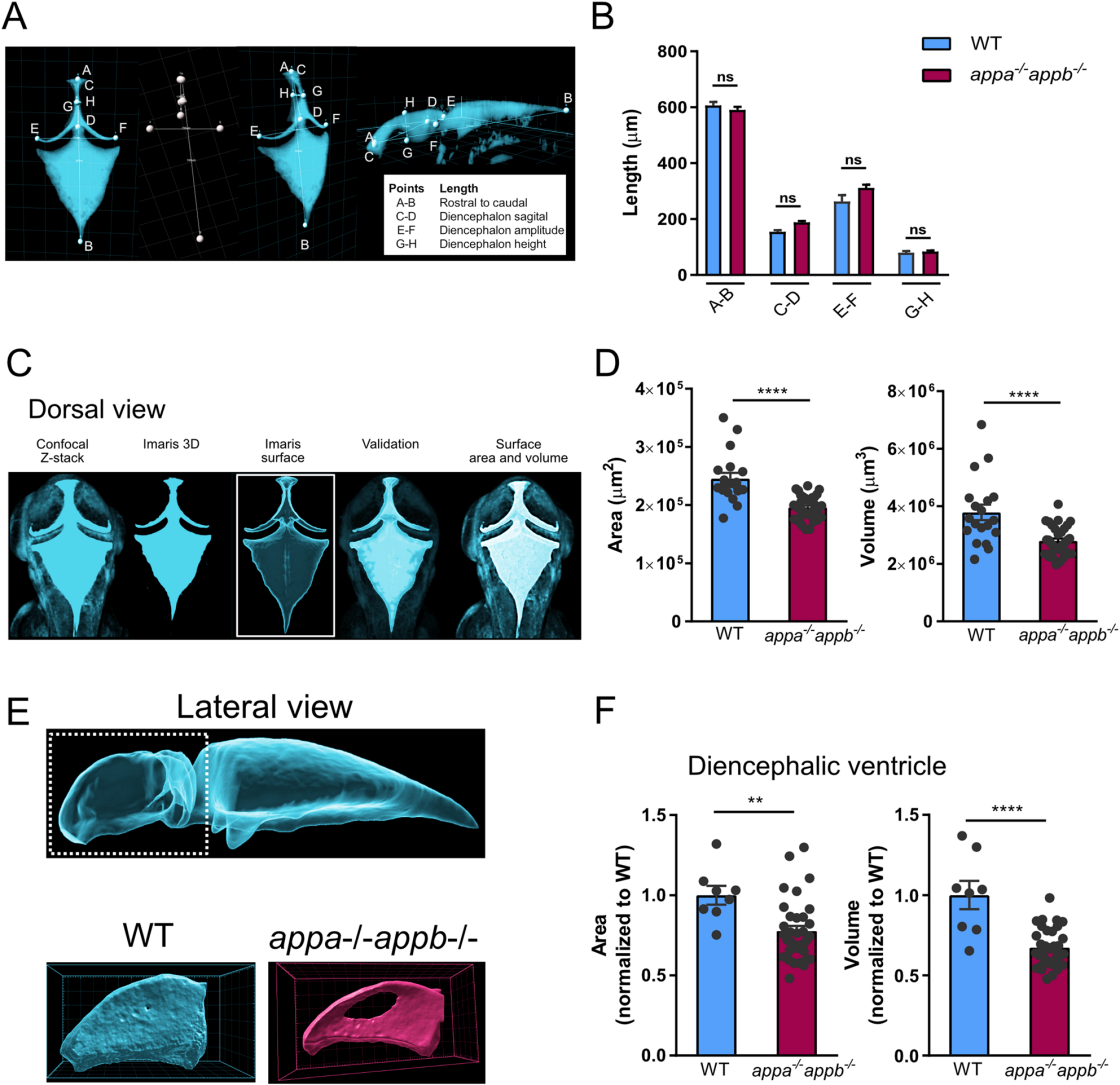
The *appa*^*−/−*^*appb*^*−/−*^ 2 dpf larvae zebrafish exhibit smaller brain ventricle. Dorsal 3D surface rending of confocal stacks taken from brain ventricles of dextran injected 2 dpf zebrafish larvae (**A**). Quantification of total ventricle surface area and volume show that both are decreased in *appa*^*-/-*^*appb*^*-/-*^ larvae (**B**). Lateral 3D surface rending of confocal stacks from brain ventricles of dextran injected 2 dpf zebrafish larvae with close up on diencephalic ventricle (**C**). Quantification of surface area and volume of the diencephalic ventricle in WT and *appa*^*-/-*^*appb*^*-/-*^ larvae (**D**). Measurement of gross ventricle morphology at 2dpf WT and *appa*^*-/-*^*appb*^*-/-*^ larvae as the length (**E**). Distance between rostral to caudal, diencephalon ventricle sagittal length, amplitude and height show no significant difference in mutants (**F**). Data are reported as mean ± SEM. ** ρ < 0.01, **** ρ < 0.001. n: (**B**) WT = 19, *appa*^*-/-*^*appb*^*-/-*^ = 34, (**D**) WT = 8, *appa*^*-/-*^*appb*^*-/-*^ = 34, (**F**) WT = 5, *appa*^*-/-*^*appb*^*-/-*^ = 4.

We then went on to address if defects in ependymal cilia affect brain ventricle formation. The gross morphology was determined by measuring the length between specific points and areas of the ventricles: rostral to caudal, diencephalon ventricle sagittal length, amplitude and height (Fig. 9A). However, no significant change was detected compared with wild-type larvae (Fig. 9B). We next analysed brain ventricle area and volume in 2dpf larvae (Fig. 9C) and found significant reductions in both area and volume of the ventricular space in *appa*^*−/−*^*appb*^*−/−*^ compared with wild-type larvae (Fig. 9D). The diencephalic ventricle was smaller in both area and volume when (Fig. 9E) compared between both genotypes (Fig. 9F). These results show that while the layout of ventricles as measured by length of App mutants is maintained, their volume are smaller.

### Ciliary targeting sequences in App

Many proteins distributed to the cilium carry one or more ciliary targeting sequences (CTS). The most common and well-studied are the VxP and AxxxQ motifs, both of which the requirement has been shown in transmembrane proteins including opsins^51–53^ and somatostatin receptor 3 (SSTR3)^54,55^. The presence of App in cilia therefore made us investigate the presence of these motifs in App. Interestingly, we found several different CTS motifs with most localized to the mid- and C-terminal domain of the App protein (Supplementary file 6). Furthermore, most of these are in conserved regions and are thus shared between zebrafish, mouse and human (Supplementary file 6).

## Discussion

In this study, we show that App localizes to different non-motile and motile cilia in zebrafish larvae including the stereo- and kinocilia of the otic vesicle, motile cilia of olfactory sensory neurons in the olfactory epithelium, and cilia of the ependymal cells lining the brain ventricles. We also show an evolutionary conserved localization of APP to cilia of the ependymal cells lining the brain ventricles of adult zebrafish, mice and humans. As these results indicated a possible function of APP in ciliogenesis or cilium function, we used zebrafish lacking the two APP orthologues, Appa and Appb, and found longer ependymal cilia and smaller brain ventricles in larvae zebrafish. Thus, our results suggest that APP not only is distributed to cilia but also seems to have an important function in ciliogenesis and brain development.

Using different antibodies, we found a punctate localization of App in cilia, indicating that the protein is randomly distributed within the cilium similar to other membrane receptors such as SSTR3 and Smoothened (Smo)^56,57^. In contrast, we observed a continuous rather than punctate localization of APP in human ependymal cilia suggesting that the distribution of APP may differ between species. The cilium membrane, although continuous with the plasma membrane, possesses a specific and conserved composition of proteins and lipids. This specification is established through an active transport of ciliary membrane proteins^58^ that at least partly depends on specific CTSs within proteins^59^. The presence of several such CTSs and their conservation between zebrafish, mice, and human supports a motif-based transport of APP to cilia.

APP expression by ependymal cells was first reported in rodents and humans in the late 1980s and early 1990s^44,45,60,61^. In line with these findings, we here not only confirm the expression of App in adult zebrafish ependymal cells, but also show that App localizes to ependymal motile cilia in vertebrates as far apart as zebrafish, mice, and humans. The longer but structurally normal ependymal cilia of *appa*^*−/−*^*appb*^*−/−*^ mutants suggests a role of App in ciliogenesis. However, the *appa*^*−/−*^*appb*^*−/−*^ mutants gave rise to fertile adults without phenotypic changes associated with primary cilium defects, such as curved body and hydrocephalus^62–65^. During early development, motile cilia of Kupffer’s vesicle are essential to establish laterality of organs^66,67^, while others maintain CSF flow within brain ventricles. Consequently, cilia-driven flow is crucial to form and maintain proper brain ventricles, as zebrafish, clawed frog and mouse ciliary mutants display ventricular defects^68^. Here, the normal overall profile and decreased volume of ventricles in *appa*^*−/−*^*appb*^*−/−*^ mutants indicate defects in ventricle inflation. In line with our findings, Olstad et al. recently reported that defects in motile cilia were likely to result in ventricle duct occlusion^68^. Thus, it is possible that the defective ventricle morphology observed in the *appa*^*−/−*^*appb*^*−/−*^ mutant larvae may result from changed CSF flow due to motile cilia defects.

CSF circulation is thought to play an important role in removal of waste products from the brain parenchyma^69^. Thus, subtle changes in the coordinated beating of cilia may contribute to altered CSF flow, impair clearance and hence contributes to a slow build-up of waste products over time in the aging brain. Supporting this are findings that individuals with Down syndrome, expressing approximately 50% higher levels of APP, have changed CSF flow in the lateral ventricles^70^ and develop Alzheimer’s disease at early age^71^. Although the morphology of ependymal cilia of DS brains is unknown, in vitro cell cultures show decreased primary cilium length^39^. Further studies on App’s effect on ciliary movement and CSF flow during development and aging are needed.

Analysis of APP fragments in CSF, currently used to diagnose individuals with cognitive impairments, are thought to represent pathological changes within the brain. However, protein fragments detected in CSF may not only originate from the brain parenchyma but may also be derived from APP processed within the ependymal cells and the protruding cilia as secretases needed for APP processing are present in cilia^72^. The release of APP from ependymal cells could be mediated through the release of extracellular cleavage products or by budding extracellular vesicles and ectosomes from the cilium^73^. Interestingly, APP-containing vesicles released into the CSF^74^ were found to have lower levels of APP in AD patients compared to healthy individuals^75^. Furthermore, a well-established feature of normal pressure hydrocephalus, where ciliary function is impaired^76^, is decreased CSF levels of soluble APP and Aβ, which are restored upon successful shunt treatment of the condition^77–79^. The impact of ependymal integrity and the contribution of cilium-mediated APP release need further studies but could potentially change the present interpretation of biomarkers used to assess disease progression. The extent to which APP mediate other processes requiring the ependymal cells including neurogenesis^61^ and migration of new-born neuronal cells^80^ still remain to be investigated.

Finally, accumulation of App at the root of the basal body, as observed in the olfactory sensory neurons and otic vesicle cilia in larval zebrafish, correlates with the findings reported by Yang and Li on APP enrichment along ciliary rootlets^81^. The presence of App in cilia mediating sensory input, both olfaction and hearing, opens up the possibility that sensory changes may not only result from defects in the brain regions receiving input from these organs, but could also be due to direct effects on their cilia. Thus, further studies on cilia of sensory neurons may give insights into the mechanisms resulting in the sensory deficiencies observed in AD mouse models and neurodegenerative diseases^82^.

Altogether, our data show the presence of App in various cilia and at least in the ependymal cilia, a conserved distribution across vertebrates. The evolutionary conserved CTSs of APP and its expression throughout development and aging suggest a central role of APP within the ependymal in both ciliogenesis and brain ventricle formation. Further studies are required to fully understand the impact of App in cilia in our olfactory and auditory organs and to which extent defects in ependymal cell integrity and ciliation contribute to APP-related developmental processes and disease progression.

## Methods

### Animal care and ethics statement

The zebrafish (*Danio rerio*) facilities and maintenance were approved and follow the guidelines of the Swedish National Board for Laboratory Animals. This study was approved by the Animal Ethical Committee at the University of Gothenburg. All procedures for the experiments were performed in accordance with the animal welfare guidelines of the Swedish National Board for Laboratory Animals and followed the recommendations in the ARRIVE guidelines^83^. Zebrafish were maintained in Aquatic Housing Systems (Aquaneering, San Diego, CA) at 28.5 °C, under a 14:10 h (h) light:dark cycle at the Institute of Neuroscience and Physiology, University of Gothenburg. Fish were fed twice daily a diet of live-hatched brine shrimps and Gemma fish food (Skretting, Amersfoort, Netherlands). System water was created using reverse osmosis water kept at a pH of 7.2–7.6 with NaHCO_3_ and coral sand and salt (Instant Ocean, Blacksburg, VA) to maintain the conductivity at 600μS. Breeding of fish was carried out in 1–2 L breeding tanks and embryos were collected in embryo medium (EM) (1.0 mM MgSO_4_, 0.15 mM KH_2_PO_4,_ 0.042 mM Na_2_HPO_4,_ 1 mM CaCl_2,_ 0.5 mM KCl, 15 mM NaCl, 0.7 mM NaHCO_3_) and raised in a dark incubator at 28.5 °C^84^.

The following fish lines were used in the present project; AB fish from the Zebrafish international resource centre (ZIRC) or was used for outbreeding and as wild-type background, *appb*^*26_2/26_2*^ and *appa*^*−/−*^ as described below^14^.

Human brain tissue samples were performed by Queen Square Brain Bank for Neurological Disorders, Department of Clinical and Movement Neurosciences, Institute of Neurology, University College London (UCL). Ethical approval for the use of human post-mortem samples was approved by a London Research Ethics Committee and tissue stored for research under a license from the Human Tissue Authority. Informed consent was obtained from each donor or obtained from the next of kin/or legal guardian(s) of the donors. Human brain tissues were used in accordance with the Helsinki declaration and the regional ethics committees at UCL and the University of Gothenburg have provided approval for the study.

### Mutagenesis using the CRISPR/Cas9 system

Genetic mutations in the *appa* gene were introduced using the CRISPR/Cas9 system as previously described^85^. Briefly, gRNAs were generated with a target-specific DNA oligonucleotide (Integrated DNA Technologies, Leuven, Belgium) containing a T7 promoter sequence in the 5’-end and a ‘generic’ DNA oligonucleotide for the guide RNA. The two oligonucleotides were annealed and extended with Platinum Taq DNA polymerase (Thermo Fisher Scientific, Waltham, MA), in a final concentration of 1 × buffer, 0.25 mM dNTP, 0.5 μM of each oligonucleotide and 0.04U/ul Taq with one cycle at the following temperatures (98 °C 2 min; 50 °C 10 min, 72 °C 10 min). The resulting product was analyzed on a 2.5% agarose (Roche, Basel, Switzerland) gel to confirm a single fragment of 120 basepairs (bp) and used to transcribe RNA. In vitro transcription was performed with the T7 Quick High Yield RNA Synthesis kit (New England Biolabs, Ipswich, MA) and incubated at 37 °C for 16 h. DNA template was removed with RNase Free DNase at 37 °C for 15 min. After purification with the RNA clean & concentrator-5 (Zymo Research, Irvine, CA), gRNA was analyzed on a 2.5% agarose gel for integrity and diluted to 250 μg/μl with RNase free water and stored at − 80 °C. Cas9 protein was diluted to 500 nM in Hepes (20 mM HEPES, pH7.5; 150 mM KCl) and stored at − 80 °C. Embryos were co-injected with 50 pg gRNA and 300 pg Cas9 protein at the one to two cell stage using a microinjector apparatus FemtoJet express (Eppendorf AG, Hamburg, Germany). Injected embryos were screened for gRNA activity using the T7 endonuclease assay (New England Biolabs, Ipswich, MA). Ten embryos from each gRNA injection were pooled at 48 hpf and genomic DNA extracted with 50 mM NaOH at 95 °C for 30 min. M13- and PIG-tailed primers (IDT, Leuven, Belgium) were used to amplify a region surrounding the mutated site of each locus using 1 × buffer, 2.5 mM MgCl_2_, 0.2 mM dNTP, 0.2 μM primers, 1U Taq polymerase (Promega, Fitchburg, WI). The polymerase chain reaction (PCR) was separated on a 1% agarose gel with the QIAquick Gel Extraction Kit (Quiagen, Hilden, Germany), and then 200 ng of the purified PCR product was dissociated and reannealed (95 °C for 5 min, 95–85 °C at − 2 °C /s, 85–25 °C at 0.1 °C /s) in a reaction containing 1 × NEB buffer 2 (New England Biolabs, Ipswich, MA) and then digested with 5U T7 endonuclease I (New England Biolabs, Ipswich, MA) for one hour at 37 °C. Fragments were analyzed on a 2% agarose gel. The remaining embryos were raised to adulthood and outcrossed with AB wild-type fish. Sixteen embryos from each outcrossed pair were screened for mutations in the F1 generation using a three-primer fluorescence PCR method. A 300–450 bp region surrounding the target site was amplified using forward primers linked with a M13 sequence and a PIG-tailed reverse primer in combination with a generic M13-FAM primer. The *appa*^*C21_16*^ mutants, refer to as *appa*^*−/−*^, carry a deletion of 10 bp in exon 2. Sanger sequencing with BigDye Terminator v1.1 Cycle Sequencing Kit (Applied Biosystems, Waltham, MA) on an ABI3130xl sequencer (SeqGen Inc, Los Angeles, CA) revealed a deletion of ten nucleotides in exon 2 that likely introduce a frameshift mutations. Heterozygous mutant carriers were raised and subsequently outcrossed into the wild-type AB fish line until generation F4. Outcrossed adults were genotyped using M13-FAM primers and PCR reactions diluted in HiDi formamide (Applied Biosystems, Waltham, MA) with ROX 500 dye size ladder (Thermo Fisher Scientific, Waltham, MA) and analyzed for amplified fragment length polymorphism (AFLP) on an ABI3130xl sequencer. Offspring from heterozygous F4 inbreeds were inbred to generate homozygous wild-type and mutant lines. Generation of *appa*^*−/−*^*appb*^*−/−*^ double mutants were obtain from mating single mutant *appa*^*−/−*^ with single mutant *appb*^*−/−*^.

### Protein sequence alignment

Sequences of APP were obtained from the UniProt database^86^ and aligned with ClustalW using MegAlign Pro v17.2.1 (DNAstar, Inc., Madison, WI) The following sequences were used; *Homo sapiens* APP751 (P05067-8), *Mus musculus* APP751 (P12023-3), *Danio rerio* Appa738 (Q90W28), Appb751 (B0V0E5). Amino acids conserved across all species were marked with bright blue background.

### Whole-mount fluorescent in situ hybridization

To detect *appa* and *appb* mRNA expression pattern in zebrafish larvae, fluorescent in situ hybridization was performed. Antisense digoxigenin-labeled *appa* and *appb* RNA probes used are described previously^14^. Zebrafish embryos were staged according to Kimmel et al. to the hours post-fertilization (hpf)^87^ and manually dechorionated with forceps (Dumont, Montignez, Switzerland). A treatment with 0.003% PTU (1- phenyl-2-thiourea) (Sigma, St. Louis, MO) was performed around 23hpf stage to prevent pigmentation. Fluorescent in situ hybridization was performed as described by Lauter et al.^88^. Briefly, zebrafish larvae were euthanized in 0.2 mg/ml ethyl 3-aminobenzoate methanesulfonate (tricaine) (MS-222, Sigma, St. Louis, MO)^84^ and fixed at 30 hpf in 4% paraformaldehyde (PFA) (Sigma, St. Louis, MO) for 24 h at 4 °C. Embryos were washed in phosphate-buffered saline (PBS) with 0.1%Tween-20 (PBST) and dehydrate into increasing methanol (MeOH) gradients from 25 to 100%. Embryos were incubated in 2% hydrogen peroxide (H_2_O_2_) for 20 min, then gradually rehydrated with decreasing MeOH gradients. Embryos were incubated in 10 μg/ml proteinase K (in 10 mM Tris–HCl pH 8.0, 1.0 mM EDTA) for 10 min at room temperature (RT). The reaction was stopped with 2 mg/ml glycine in PBST and then the embryos were postfix in 4.0% PFA for 20 min. PBST washes were performed before incubation in prehybridization buffer (HB; 50% deionized formamide, 5 × saline-sodium citrate (SSC) (3 M NaCl, 300 mM tri-sodium citrate, pH 7.0), 5 mg/ml torula RNA (Sigma, St. Louis, MO), 50 μg/ml heparin sodium salt and 0.1% Tween-20). Embryos were pre-hybridized at 70 °C for 1 h. Then, hybridization was done with selectively 50 ng of DIG-labelled *appa* or *appb* RNA in HB with 5% dextran sulfate (Sigma, St. Louis, MO) at 70 °C overnight. The next day, embryos were washed in warm SSC with 0.1% Tween-20 followed by PBST only. After that, a 1 h-blocking incubation at RT in PBST with 8% goat serum (Sigma, St. Louis, MO) was performed. For the antibody treatment, a sheep-anti-digoxigenin-peroxidase (POD)-Fab fragments antibody (1:500 in blocking solution) (Roche, Basel, Switzerland) was used and embryos were incubated in the dark overnight at 4 °C, without agitation. To remove excess antibody, embryos were then washed in PBST at RT in gentle agitation. To amplify the signal, tyramide signal amplification (TSA) was used by combining 5-carboxyfluorescein succinimidyl ester (Molecular Probes, Eugene, OR) with tyramine hydrochlorine (Sigma, St. Louis, MO) at a 1.1:1 respective equimolar ratio. Vanillin (0.45 mg/mL) (Sigma, St. Louis, MO) was used as a POD accelerator and diluted in borate buffer pH 8.5. Embryos were incubated with the TSA and POD accelerator reaction in the dark without agitation for 15 min at RT. To stop the TSA reaction, embryos were washed in PBST and then incubate in 100 mM glycine–HCl pH 2.0 to inactivate the POD reaction followed by additional PBST washing. To avoid shrinkage, embryos were then incubated in an increasing glycerol gradient (in PBST, 40 mM NaHCO_3_). Whole embryos were mounted on glass bottom 35 mm Petri dish (Cellvis, Mountain View, CA) in 1% low-melting agarose (Sigma, St. Louis, MO). Samples were imaged as stacks using inverted Nikon A1 confocal system (Nikon Instruments, Melville, NY) using a 20 × objective (Plan-Apochromat 20×/0,75) and 40 × water-immersion objective (Apochromat LWD 40×/1,15). Image processing was done using ImageJ FIJI software (NIH, Bethesda, MD).

### Immunofluorescence

#### Zebrafish larvae

To detect protein expression, immunofluorescence experiments were performed in whole-mount AB zebrafish larvae. A treatment with 0.003% PTU was performed around 23hpf stage to prevent pigmentation. Then freshly euthanized embryos were fixed at 30 hpf for 2 h in 4% PFA at RT on slow agitation. After fixation, embryos were washed with PBS with 0.5% Triton X-100 (PBTx) at RT. Followed up by incubation in blocking solution (5% goat serum donor herd (GS) (Sigma, St. Louis, MO), 2% bovine serum albumin (BSA) (Sigma, St. Louis, MO), 1% DMSO (Sigma, St. Louis, MO) and 0.5% PBTx) for 3 h at RT. The larvae were then incubated overnight at 4 °C on slow agitation with the desired primary antibodies in blocking solution: mouse IgG2b anti-acetylated tubulin monoclonal antibody (1:1000) (Sigma, St. Louis, MO), recombinant rabbit anti-amyloid precursor protein monoclonal antibody Y188 (1:500) (Abcam, Cambridge, United Kingdom), and/or mouse anti-glutamylated tubulin monoclonal antibody (1:1000) (Adipogen, San Diego, CA). The zebrafish larvae used for negative control were incubated in blocking solution only. The next day, embryos were washed (5 × 45 min) with PBSTx at RT and incubated in dark with the specific secondary antibodies overnight at 4 °C, in blocking solution: goat anti-mouse IgG2b Alexa Fluor-647 (1:1000) (Thermo Fisher Scientific, Waltham, MA) and goat anti-rabbit IgG Alexa Fluor-488 (1:1000) (Thermo Fisher Scientific, Waltham, MA), or goat anti-mouse IgG1 Alexa Fluor-568 (1:1000) (Thermo Fisher Scientific, Waltham, MA). The zebrafish larvae used for negative control were also incubated with the former secondary antibodies. The larvae were then washed with PBTx at RT and incubated for 15 min with DAPI (1:1000) (Thermo Fisher Scientific, Waltham, MA) to stain the nuclei in PBS at RT before the final washes. Stained larvae were mounted in 1% low-melting point agarose, on glass bottom 35 mm Petri dish.

#### Adult zebrafish and mouse brains

Brains from adult zebrafish (AB, 2 year-old) and mouse (C57Bl6/n, 8–9 week-old). Brains were fixed in 4% PFA in PBS overnight at 4 °C and then washed and immersed in 30% sucrose solution in PBS, after which they were frozen in OCT cryomount (Histolab, Askim, Sweden). Coronal or sagittal cryosections from adult zebrafish (25 μm) and mouse brains (16 μm) slices were stored at − 80 °C prior to use. Sections were air dried for 15 min at RT then rehydrated in PBS. Slices were permeabilized in 0.1% PBTx for 10 min at RT and washed 3 × in PBS for 15 min each. A 0.1% Sudan Black B (SBB) (Sigma, St. Louis, MO) in 70% EtOH treatment was performed for 20 min at RT. Slides were then washed in PBS for 3 × 5 min. The slides were then incubated in blocking solution of 2% GS in PBS at RT for 1 h, followed by the incubation with the primary antibodies in 2% BSA at 4 °C overnight: mouse IgG2b anti-acetylated tubulin monoclonal antibody (1:1000), recombinant rabbit anti-amyloid precursor protein monoclonal antibody (Y188) (1:500) or mouse anti-amyloid precursor protein A4 antibody (clone 22C11) (1:500) (Merck Millipore, Burlington, MA), or rabbit IgG (1:500) (Abcam, Cambridge, United Kingdom) and/or with blocking solution only for negative controls. The next day, slides were washed 3 × in PBS for 15 min each and incubated with the secondary antibody in 2% BSA at RT for 3.5 h combined with DAPI (1:1000): goat anti-mouse IgG2b Alexa Fluor-647 (1:1000) and/or goat-anti rabbit Alexa Fluor-488 (1:1000) and/or goat anti-mouse IgG1 Alexa Fluor-488 (1:1000) (Thermo Fisher Scientific, Waltham, MA) and/or goat anti-mouse IgG1 Alexa Fluor-568 (1:1000). The slides were then washed 3 × 15 min in PBS and shortly rinsed in ddH_2_O to remove any residual salts. The slides were covered with coverslips using ProLong gold antifade mounting medium (Thermo Fisher Scientific, Waltham, MA).

Samples were imaged using Zeiss LSM710 inverted confocal microscope (Carl-Zeiss, Jena, Germany) using 40 × water immersion objective (Plan-Apochromat 40×/1.0) and a 63 × oil-immersion objective (Plan-Apochromat 40×/1.0) or with Zeiss LSM880 Airyscan inverted confocal microscope (Carl-Zeiss, Jena, Germany) using 40 × water immersion objective (LCD-Apochromat 40×/1.0) and 63 × oil-immersion objective (Plan-Apochromat 63x/1.4). Image processing and intensity profiles were performed with ImageJ FIJI program.

### Immunostaining of human brain sections

Neurologically normal human post-mortem control tissue was obtained from Queen Square Brain Bank for Neurological Studies. Paraffin-embedded sections were cut from caudate nucleus brain region, which contains ependymal lining containing cilia. Sections were dewaxed in three changes of xylene and rehydrated using graded alcohols. Endogenous peroxidase activity was blocked using 0.3% H_2_O_2_ in MeOH for 10 min followed by pressure cooker pre-treatment for 10 min in citrate buffer, pH 6.0. Non-specific binding was blocked using 10% non-fat dried milk (Sigma-Aldrich, St. Louis, MO) in Tris buffered saline-Tween (TBS-Tween) before incubating with either anti-acetylated tubulin (1:1000) or anti-APP (1:500) antibodies at RT for 1 h. A biotinylated mouse anti-rabbit IgG antibody (1:200) (Agilent DAKO, Glostrup, Denmark) was added for a 30 min incubation with the sections at RT followed by avidin–biotin complex (Vector Laboratories, Burlingame, CA). Coloration was developed with di-aminobenzidine (Sigma-Aldrich, St. Louis, MO) activated with H_2_O_2_^89^.

### Protein extraction from whole zebrafish larvae and western blotting

Protein was extracted from 3dpf double *appa*^*−/−*^*appb*^*−/−*^ mutant whole larvae (60 larvae per n, n = 3) to confirm loss of protein. Larvae were euthanized, deyolked with ice-cold PBS and snap frozen in liquid nitrogen prior to use and stored at − 80 °C. Samples were homogenized in an ice-cold lysis buffer (10 mM Tris–HCl pH 8.0, 2% sodium deoxycholate, 2% SDS, 1 mM EDTA, 0.5 M NaCl, 15% glycerol) supplemented with protease inhibitors cocktail (Roche, Basel, Switzerland) and using glass tissue grinder, on ice. Samples were then incubated 20 min on ice, sonicated for 10 min on max level and centrifuged at 10,000×*g* at 4 °C. Supernatants were collected and kept on ice and protein concentration measured with a BCA Protein Assay Kit (Thermo Fisher Scientific, Waltham, MA) and samples stored at − 80 °C. Proteins samples (40-60ug) were then diluted in a denaturing lysis buffer (1X NuPAGE LDS Sample Buffer (Thermo Fisher Scientific, Waltham, MA), 0.05 M DTT (Sigma-Aldrich, St. Louis, MO), lysis buffer completed with protease inhibitors) and then boiled for 5 min at 95 °C. Proteins were then separated on a NuPAGE NOVEX Bis–TRIS pre-cast gel (Thermo Fisher Scientific, Waltham, MA) and transferred onto a 0.2 μm nitrocellulose membrane (GE Healthcare, Chicago, IL). The membrane was incubated in a blocking solution (5% milk) for 2 h at RT and then immunoblotted with the desired primary antibodies overnight at 4 °C: rabbit anti-amyloid precursor protein monoclonal antibody (Y188) (1:2000) or mouse anti-amyloid precursor protein A4 antibody (clone 22C11) (1:5000) and with a loading concentration control mouse anti-GAPDH-HRP conjugated (1:20,000) (Novus Biologicals, Centennial, CO) or mouse anti-α-tubulin monoclonal (1:10,000) (Sigma, St. Louis, MO). The membrane was then washed in TBS-Tween 3 × 10 min at RT and incubated with the secondary antibodies anti-rabbit-HRP (1:5000) (Cell Signaling, Danvers, MA) for 1 h at RT. The membrane was washed 3 × 10 min in TBS-Tween before being developed. The signal was developed using SuperSignal West Dura Extended Duration Substrate kit (Thermo Fisher Scientific, Waltham, MA) and imaged using ChemiDoc Imaging (Bio-Rad, Hercules, CA). Western blot images were processed and analysed using Image Lab program (Bio-Rad, Hercules, CA). Quantification of band intensities were performed by Image Lab (Bio-Rad, Hercules, CA) with GAPDH or alpha-tubulin used to control protein loading. Samples were normalized to controls.

### RNA extraction from whole zebrafish larvae and qPCR

To confirm *appa* and *appb* mRNA levels decrease in our double mutant (*appa*^*−/−*^*appb*^*−/−*^), RNA was extracted from 24 hpf whole larvae (10 larvae per n, n = 5). Total RNA was extracted using TRI Reagent (Sigma, St. Louis, MO). Then, RNA samples were treated with RQ1 RNase-free DNase 1 × reaction buffer and RQ1 RNase-free DNase (Promega, Fitchburg, WI). cDNA was synthesized using High-Capacity RNA-to-cDNA Kit (Applied Biosystems, Waltham, MA) with RNase inhibitor and converted in a single-cycle reaction on a 2720 Thermal Cycler (Applied Biosystems, Waltham, MA). Quantitative PCR was performed with inventoried TaqMan Gene Expression Assays with FAM reporter dye in TaqMan Universal PCR Master Mix with UNG (Thermo Fisher Scientific, Waltham, MA). The assay was carried out on Micro-Amp 96-well optical microtiter plates (Thermo Fisher Scientific, Waltham, MA) on a 7900HT Fast QPCR System (Applied Biosystems, Waltham, MA). qPCR results were analysed with the SDS 2.3 software (Applied Biosystems, Waltham, MA). cDNA values from each sample was normalized with average C_T_’s of house-keeping genes (*eef1a1l1* and *actb1*), then the relative quantity was determined using the ΔΔC_T_ method^90^ with the sample of wild-type sibling embryos (24 hpf) as the calibrator. TaqMan Gene Expression Assays (Applied Biosystems, Waltham, MA) were used for the following genes: amyloid beta (A4) precursor protein A (*appa*) (Dr03144365_m1), eukaryotic translation elongation factor 1 alpha 1, like 1 (*eef1a1l1*) (Dr03432748_m1) and actin, beta 1 (*actb1*) (Dr03432610_m1).

### Cilium length measurement in zebrafish larvae

To compare the number of brain ependymal cilia and their length, 30 hpf AB wild-type and *appa*^*−/−*^*appb*^*−/−*^ zebrafish larvae were used. The larvae were treated with PTU, fixed in 4% PFA and the immunostaining with antibody against acetylated tubulin was performed as describe in the section above. Stacks (of around 25 μm depending on the angle of the mounted sample) were taken in the region of interest (ROI) of the dorsal portion of the diencephalic ventricle using Zeiss LSM710 confocal microscope using inverted 40 × water immersion objective (Plan-Apochromat 40×/1.0). Images were then processed using Imaris (Bitplane, Belfast, United Kingdom) and the cilium length was measured with the acetylated tubulin signal using the ¨*measuring points*¨ tool of the program. Raw data of the measurement were exported to Microsoft Excel and compiled into GraphPad Prism 7 for statistical analysis.

### Brain ventricles injection and size measurement

To measure the size of the brain ventricles in live zebrafish, 2dpf PTU-treated zebrafish larvae were used. Rhodamine-Dextran injection protocol was performed as describe by Gutzman and Sive^91^. Briefly, the larvae were anesthetized with tricaine in the EM and transferred onto a Petri dish covered with 1% agarose, lined with rows moulded. The larvae were kept in EM complemented with tricaine during the whole procedure and place on a ventral position, with top of their head facing upwards. Injections were performed using borosilicate injection needles previously pulled (P-97 Flaming/Brown micropipette puller) (Sutter Instrument, Novato, CA). Using a microinjector apparatus, 2 nl of Rhodamine B isothiocyanate-Dextran (Sigma, St. Louis, MO) were injected in the hindbrain ventricle without perforating or hitting the brain tissue below.

Larvae with non-effective injections were sorted out using a fluorescent stereomicroscope (Nikon Instruments, Melville, NY). Quickly after the sorting, the larvae were mounted in 1% low-melting point agarose on glass bottom 35 mm Petri dish. Confocal imaging stacks were acquired using an inverted Nikon A1 confocal system using a 20 × objective (Plan-Apochromat 20×/0,75). Image processing of the confocal stacks were done with Imaris program. The “*surface*” tool option was used for each sample. Data of the surface volume and area were automatically generated by the program. Length measurements of the areas of the ventricles were obtain manually with the “*measuring tool*”. All data were exported into Microsoft Excel and GraphPad 7 Prism for statistical analysis.

### Transmission electron microscopy

To evaluate the integrity of the internal structure of the axonemes and microtubules doublets of the brain motile cilia in older zebrafish, transmission electron microscopy was performed on fixed brains. Adult zebrafish were euthanized in tricaine and brains dissected, rinsed in ice-cold PBS and fixed in 2% PFA and 2% glutaraldehyde (Sigma, St. Louis, MO), in 0.042 M Millonig buffer (0.081 M Na_2_HPO_4_, 0.0183 M NaH_2_PO_4_, 0.086 M NaCl) pH 7.4 at least 24 h at 4 °C. After fixation, brains were cut in two halves and then treated in 2% osmium tetroxide (Sigma, St. Louis, MO) in 0.1 M Millonig buffer pH 7.4. Specimens were then rinsed and incubated overnight in 4% sucrose solution in 0.1 M Millonig buffer pH 7.4 after which they were dehydrated in series of ethanol and embedded in a mix of acetone and agar 100 resin plastic (TAAB Laboratories Equipment Ltd, Berks, United Kingdom) and allowed to polymerize for 48 h. Blocks were trimmed as semi-thin (1 μm) and ultra-thin (70 nm) sections collected with a commercial ultramicrotome (Leica EM UC7, Leica Microsystems, Wetzlar, Germany). Sections were post-stained with 5% uranyl acetate in distilled H_2_O during 40–60 min, rinsed in distilled H_2_O and then treated with 0.3% Lead Citrate (Thermo Fisher Scientific, Waltham, MA) for 30–60 s. Images were acquired using secondary electron detection. Images were acquired with a Tecnai Spirit BT transmission electron microscope (Field Electron and Ion Company, Hillsboro, OR).

### Statistical analysis

Statistical analysis was performed using GraphPad 7 software (Prism, San Diego, CA). Data were presented as means with standard deviation (± SD) or standard errors of the mean (± SEM). For analysis of cilium length, D’Agostino & Pearson normality test (P < 0.0001) and non-parametric two-tailed Mann–Whitney U tests were performed. Results related to qPCR and western blot quantification, and ventricle size measurements were compared statistically using unpaired Student’s t-tests. Statistical significance was set at ρ < 0.05 (*), 0.01 (**), 0.005 (***) and 0.0001 (****).

## Supplementary Information

Supplementary Information 1.

Supplementary Information 2.

## Data Availability

The datasets generated during the current study are available in the supplementary data file.
