# Supplementary material for: The localization of amyloid precursor protein to ependymal cilia in vertebrates and its role in ciliogenesis and brain development in zebrafish
Source: Sci Rep. 2021 Sep 27;11:19115. doi: 10.1038/s41598-021-98487-7 (PMC8476544; doi:10.1038/s41598-021-98487-7)
Supplement: Supplementary file 2 — Supplementary Information 2. [file 41598_2021_98487_MOESM2_ESM.docx]

Supplementary files

**Supplementary file 1.** Negative control for whole-mount fluorescent *in situ*

**
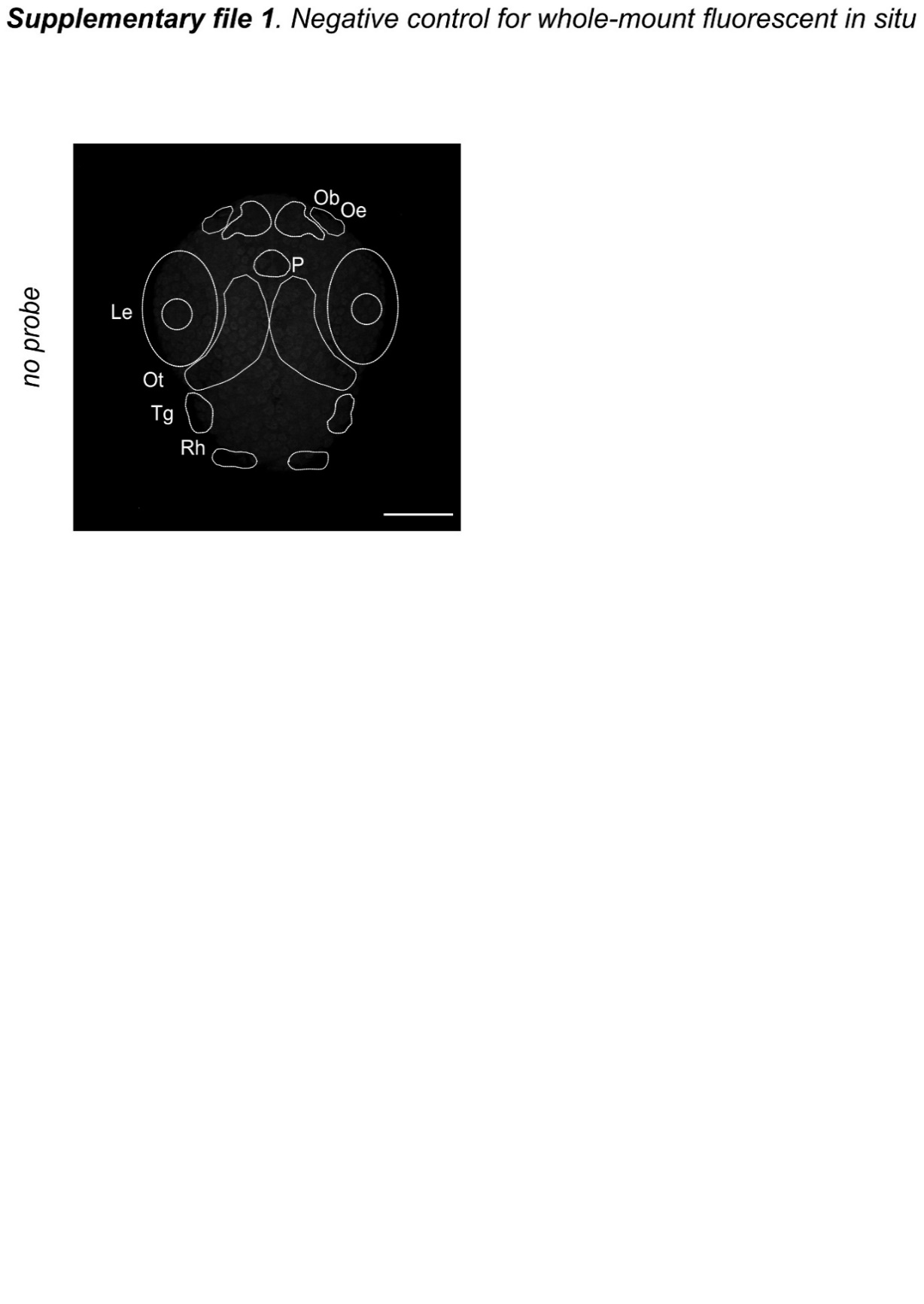
**

**Supplementary file 1. Negative control for whole-mount fluorescent *in situ*.** Whole-mount fluorescent *in situ* in the absence of mRNA probe in 30 hpf WT larvae zebrafish. Maximum projection (77 stacks). T= telencephalic ventricle, D/M= diencephalic/mesencephalic ventricle, R= rhombencephalic ventricle, Ob= olfactory bulb, Oe= olfactory epithelium, P= pituitary gland, Le= lens, Ot= optic tectum, Tg= trigeminal ganglia, Rh= rhombomeres. Magnification: 20x. Scale bar: 100µm.


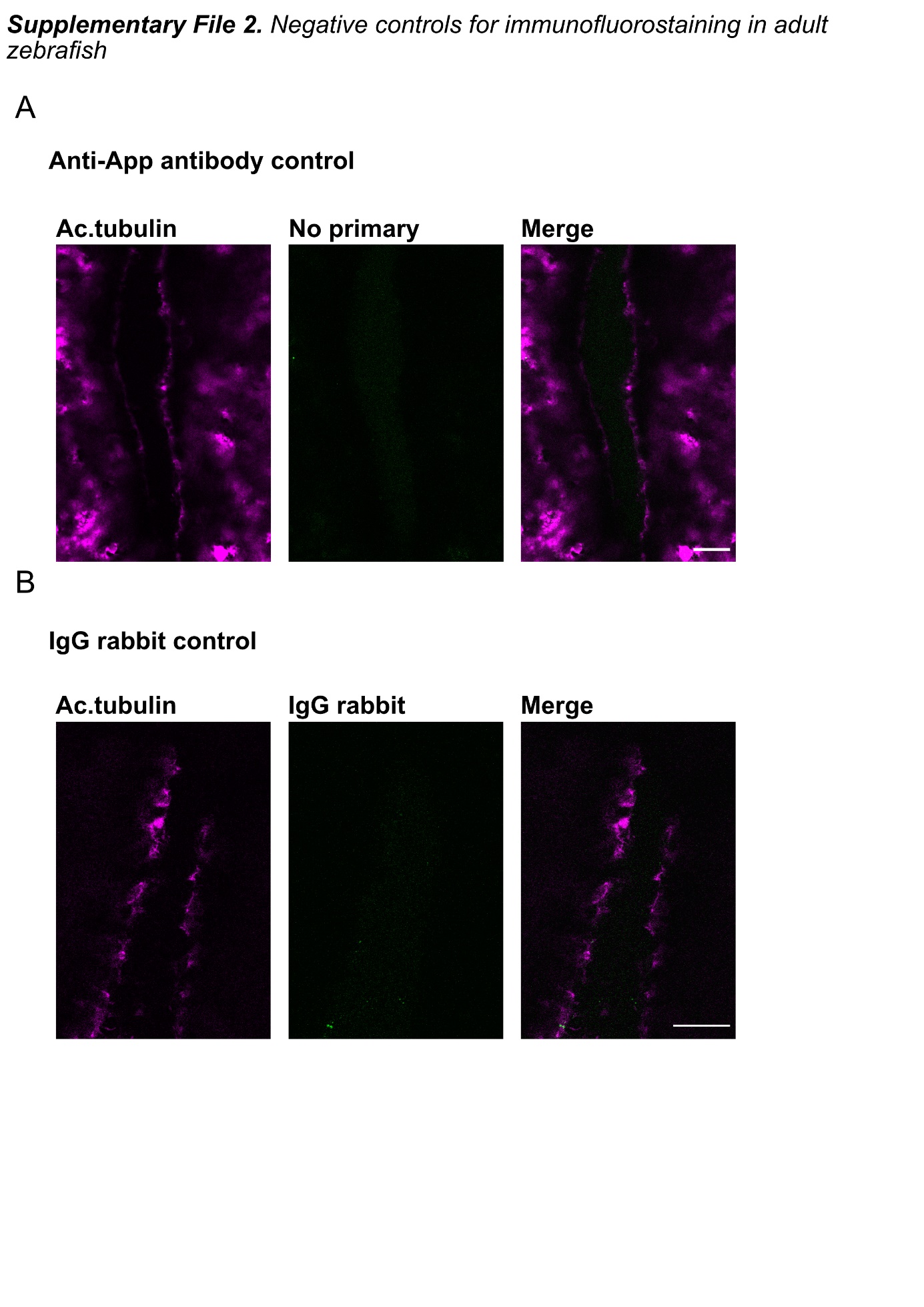
**Supplementary file 2.** Negative controls for immunofluorostaining in adult zebrafish

**Supplementary file 2. Negative immunofluorescence control in adult zebrafish.** Adult zebrafish brain slices stained with anti-acetylated tubulin antibody and (**A**) secondary anti-rabbit Alexa488 antibody (without anti-App (Y188) antibody) or with (**B**) rabbit IgG serum and secondary anti-rabbit Alexa488 antibody. Magnification: (**A-B**)= 60x. Scale bar: (**A-B**)= 20µm.


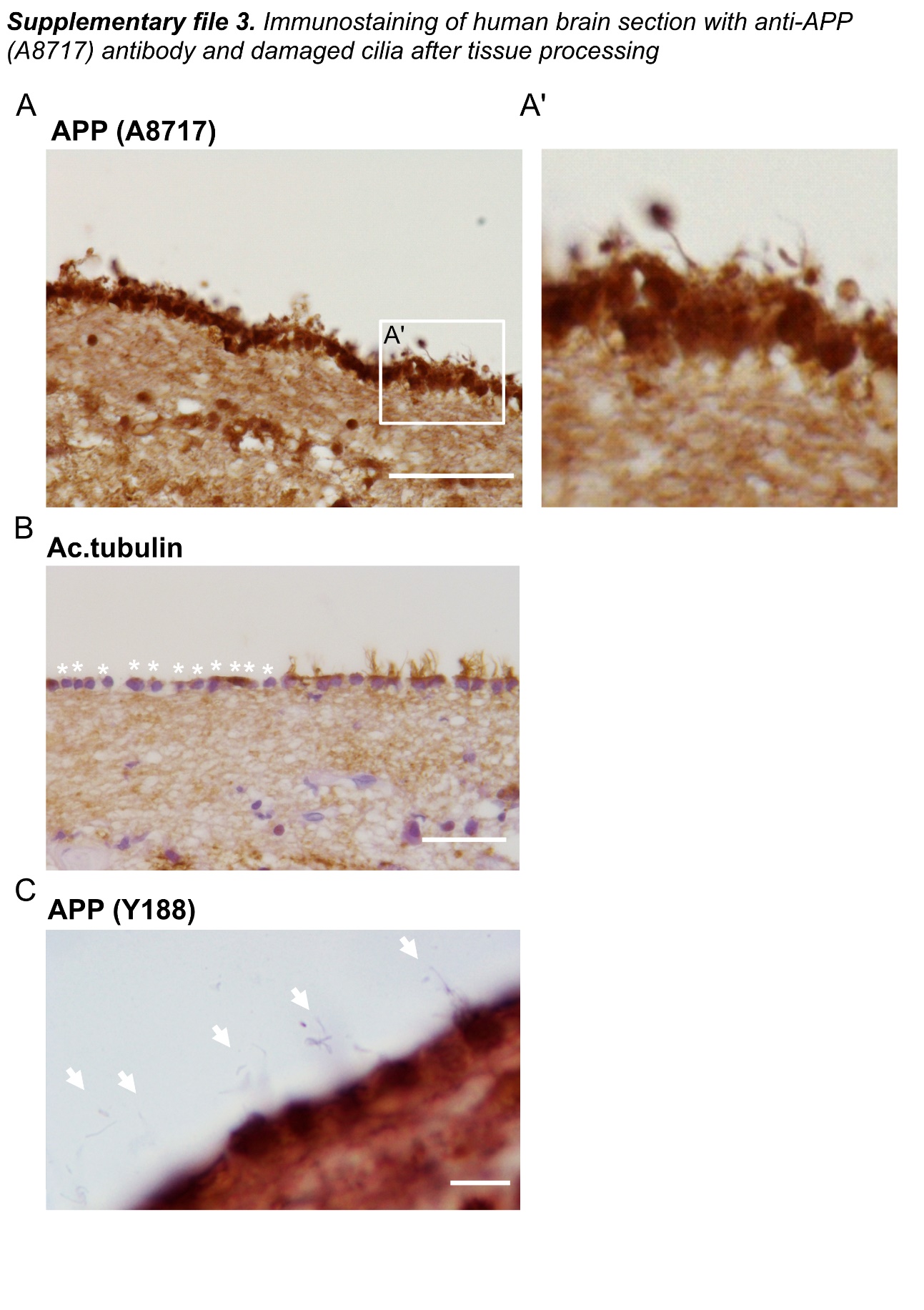
**Supplementary file 3.** Immunostaining of human brain section with anti-APP (A8717) antibody and damaged cilia after tissue processing

**Supplementary file 3. Immunohistochemical staining of APP in human brain section**. (**A**) Detection of APP with anti-APP 8A717 confirms the accumulation of APP within ependymal cells and along ependymal cilia. Close up in (**A’**). (**B**,**C**) Sections immunostained with an anti-acetylated tubulin (**B**) or an anti-APP (Y188) (**C**) antibody reveal that whereas some cilia seem to remain intact, some are completely damaged (see asterisks). White arrows indicate portions of cilia detached from their ependymal cells (**C**). Magnification: (**A,B**)= 40x, (**C**)= 100x. Scale bar: (**A**)= 50 µm, (**B**)= 10µm, (**C**)= 2µm.

**Supplementary file 4.** Full-size western blots of 22C11, Y188 and alpha-tubulin


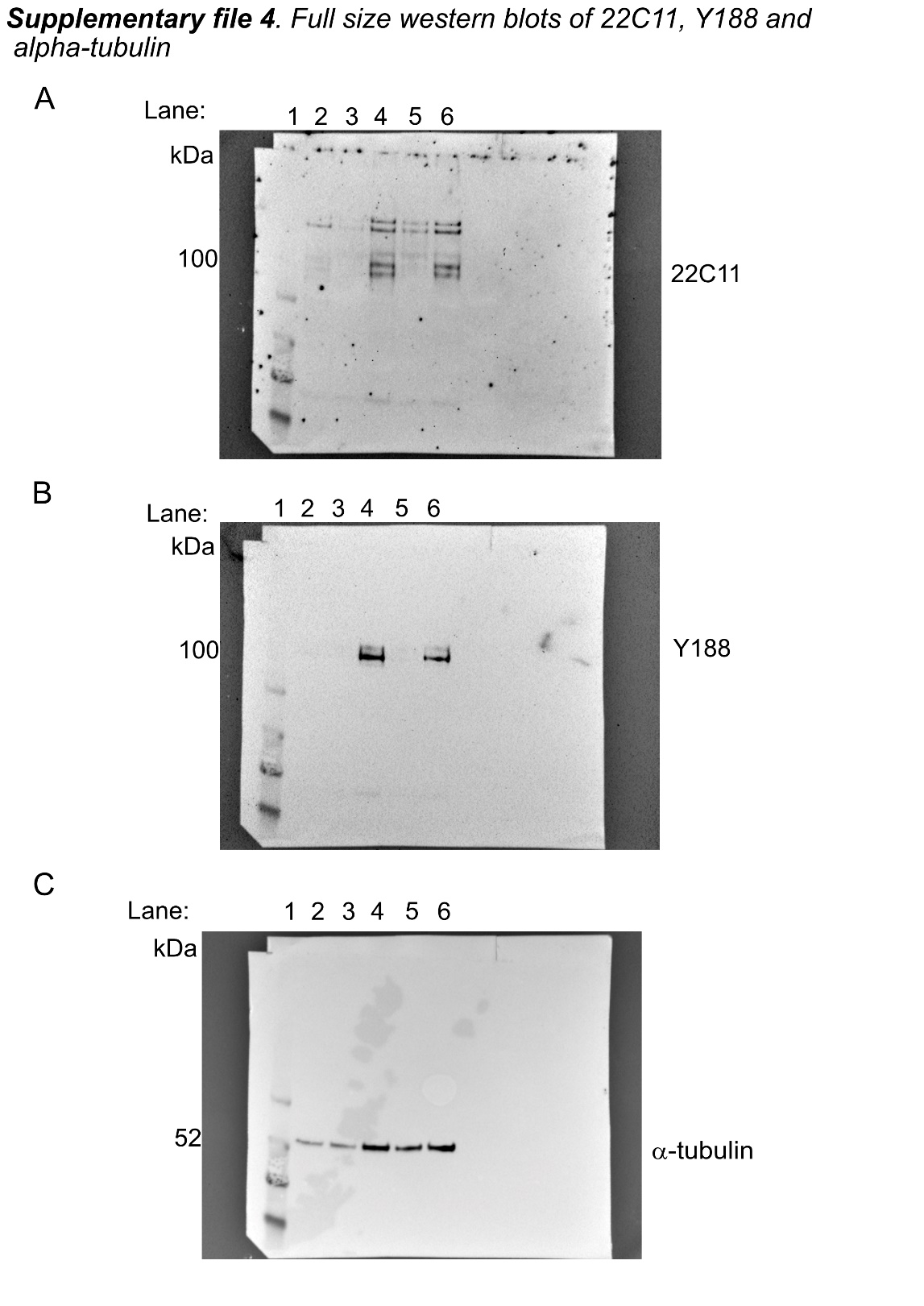


**Supplementary file 4**. **Full size western blots of 22C11, Y188 and alpha-tubulin of 3 dpf whole larvae zebrafish.** Whole western blots of 3 dpf whole larvae zebrafish with antibodies against (**A**) 22C11, (**B**) Y188 and (**C**) α-tubulin. α-tubulin was used as loading control. Lane 1: See blue2 ladder, Lanes 2,4,6: wild-type, Lanes 3,5: *appa^-/-^appb^-/-^.* Same original gel.

**Supplementary file 5.** Frequency distribution of the length of brain ventricle in 30 hpf larvae zebrafish

**
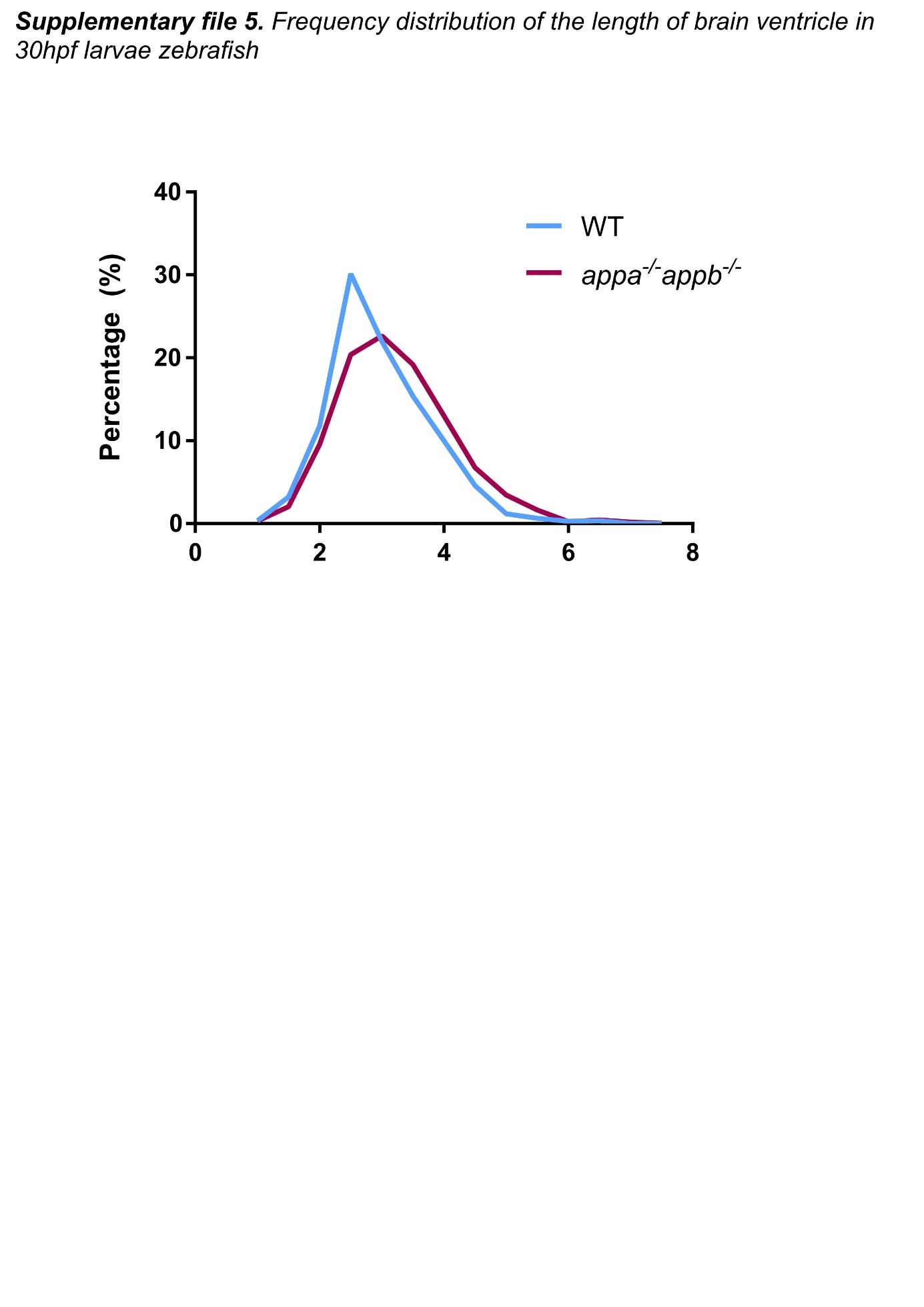
**

**Supplementary file 5**. **Frequency distribution of cilia length in 30hpf larvae zebrafish diencephalic/mesencephalic ventricle.** A higher percentage of smaller cilia in WT (blue curve) compared to *appa^-/-^appb^-/-^* cilia population (magenta curve). n=10 WT (1091 cilia), n=16 *appa^-/-^appb^-/-^* (1511 cilia).

**Supplementary file 6.** Ciliary targeting sequences in human, mouse and zebrafish
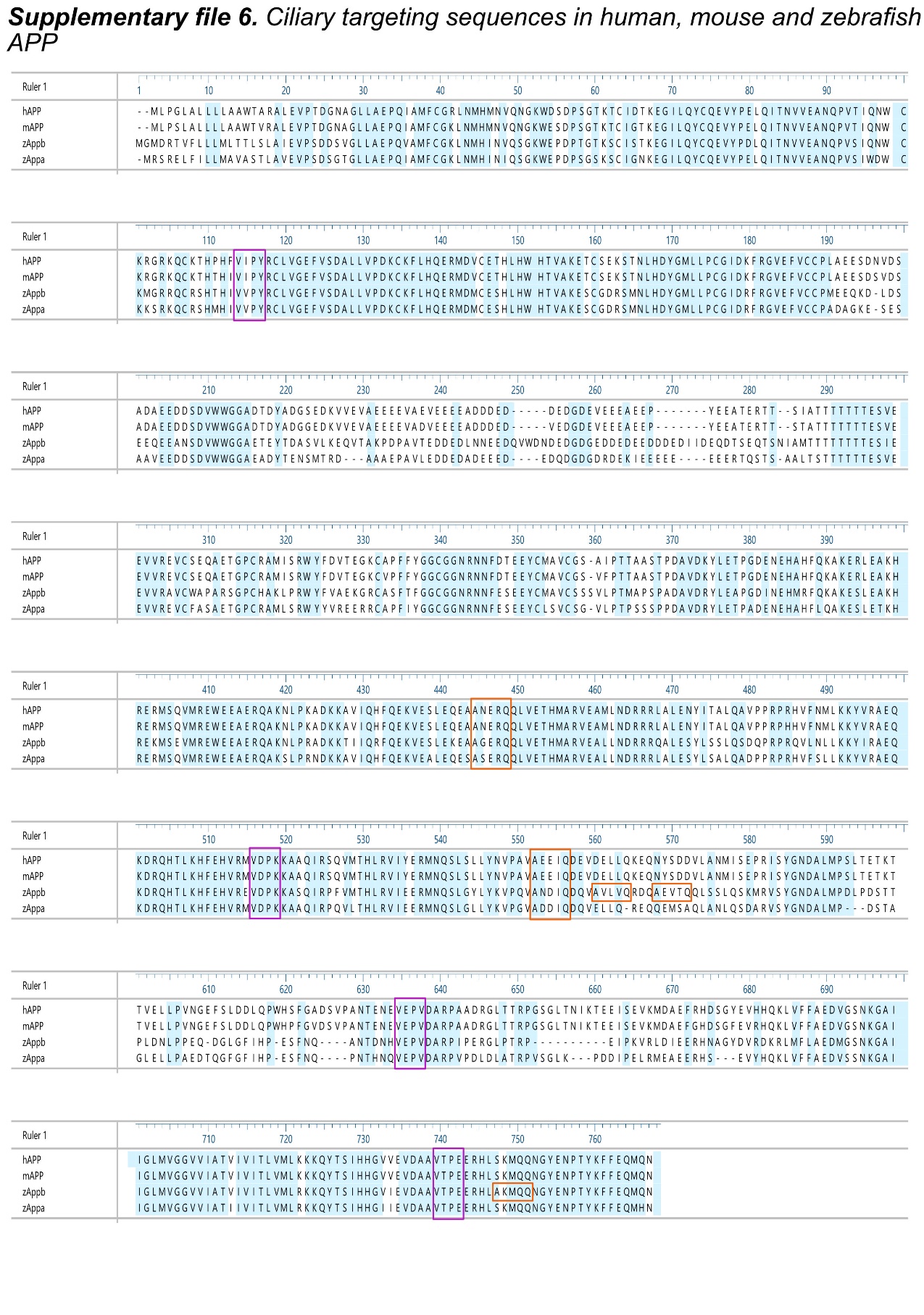


**Supplementary file 6.** **Ciliary targeting sequences in human, mouse and zebrafish APP.** Proteins sequence alignment of human APP751, mouse APP751 and zebrafish Appa738 and Appb751. Bright blue background shows conserved amino acids between species. Ciliary targeting sequences AxxxQ (orange boxes) and VxPx (purple boxes).
